# Electronic data collection in a multi-site population-based survey: EN-INDEPTH study

**DOI:** 10.1186/s12963-020-00226-z

**Published:** 2021-02-08

**Authors:** Sanne M. Thysen, Charlotte Tawiah, Hannah Blencowe, Grace Manu, Joseph Akuze, M. Moinuddin Haider, Nurul Alam, Temesgen Azemeraw Yitayew, Angela Baschieri, Gashaw A. Biks, Francis Dzabeng, Ane B. Fisker, Md. Ali Imam, Justiniano S. D. Martins, Davis Natukwatsa, Joy E. Lawn, Vladimir Sergeevich Gordeev, the Every Newborn-INDEPTH Study Collaborative Group, Peter Waiswa, Hannah Blencowe, Judith Yargawa, Joseph Akuze, Ane B. Fisker, Justiniano S. D. Martins, Amabelia Rodrigues, Sanne M. Thysen, Gashaw Andargie Biks, Solomon Mokonnen Abebe, Tadesse Awoke Ayele, Telake Azale Bisetegn, Tadess Guadu Delele, Kassahun Alemu Gelaye, Bisrat Misganaw Geremew, Lemma Derseh Gezie, Tesfahun Melese, Mezgebu Yitayal Mengistu, Adane Kebede Tesega, Temesgen Azmeraw Yitayew, Simon Kasasa, Edward Galigawango, Collins Gyezaho, Judith Kaija, Dan Kajungu, Tryphena Nareeba, Davis Natukwatsa, Valerie Tusubira, Yeetey A. K. Enuameh, Kwaku P. Asante, Francis Dzabeng, Seeba Amenga Etego, Alexander A. Manu, Grace Manu, Obed Ernest Nettey, Sam K. Newton, Seth Owusu-Agyei, Charlotte Tawiah, Charles Zandoh, Nurul Alam, Nafisa Delwar, M. Moinuddin Haider, Md Ali Imam, Kaiser Mahmud, Angela Baschieri, Simon Cousens, Vladimir S. Gordeev, Victoria Ponce Hardy, Doris Kwesiga, Kazuyo Machiyama

**Affiliations:** 1 Bandim Health Project, Bissau, Guinea-Bissau; 2 Research Centre for Vitamins and Vaccines, Statens Serum Institut, Copenhagen, Denmark; 3 Bandim Health Project, OPEN, Institute of Clinical Research, University of Southern Denmark, Odense, Denmark; 4 Kintampo Health Research Centre, Kintampo, Ghana; 5 Maternal, Adolescent, Reproductive & Child Health (MARCH) Centre, London School of Hygiene & Tropical Medicine, London, UK; 6 Dept. of Health Policy, Planning and Management, Makerere University School of Public Health, Kampala, Uganda; 7 Centre of Excellence for Maternal Newborn and Child Health Research, Makerere University, Kampala, Uganda; 8 Health Systems and Population Studies Division, icddr,b, Dhaka, Bangladesh; 9 Dabat Research Centre Health and Demographic Surveillance System, Dabat, Ethiopia; 10 Dept. of Health Services Management and Health Economics, Institute of Public Health College of Medicine and Health Sciences, University of Gondar, Gondar, Ethiopia; 11 IgangaMayuge Health and Demographic Surveillance System, Makerere University Centre for Health and Population Research, Makerere, Uganda; 12 Institute of Population Health Sciences, Queen Mary University of London, London, UK

**Keywords:** Electronic data collection, Data collection application, Field experiences, Household surveys, DHS

## Abstract

**Background:**

Electronic data collection is increasingly used for household surveys, but factors influencing design and implementation have not been widely studied. The Every Newborn-INDEPTH (EN-INDEPTH) study was a multi-site survey using electronic data collection in five INDEPTH health and demographic surveillance system sites.

**Methods:**

We described experiences and learning involved in the design and implementation of the EN-INDEPTH survey, and undertook six focus group discussions with field and research team to explore their experiences. Thematic analyses were conducted in NVivo12 using an iterative process guided by a priori themes.

**Results:**

Five steps of the process of selecting, adapting and implementing electronic data collection in the EN-INDEPTH study are described. Firstly, we reviewed possible electronic data collection platforms, and selected the World Bank’s Survey Solutions® as the most suited for the EN-INDEPTH study. Secondly, the survey questionnaire was coded and translated into local languages, and further context-specific adaptations were made. Thirdly, data collectors were selected and trained using standardised manual. Training varied between 4.5 and 10 days. Fourthly, instruments were piloted in the field and the questionnaires finalised. During data collection, data collectors appreciated the built-in skip patterns and error messages. Internet connection unreliability was a challenge, especially for data synchronisation. For the fifth and final step, data management and analyses, it was considered that data quality was higher and less time was spent on data cleaning. The possibility to use paradata to analyse survey timing and corrections was valued. Synchronisation and data transfer should be given special consideration.

**Conclusion:**

We synthesised experiences using electronic data collection in a multi-site household survey, including perceived advantages and challenges. Our recommendations for others considering electronic data collection include ensuring adaptations of tools to local context, piloting/refining the questionnaire in one site first, buying power banks to mitigate against power interruption and paying attention to issues such as GPS tracking and synchronisation, particularly in settings with poor internet connectivity.

## Key findings

**Table:** 

**WHAT IS NEW?**	
**• What was known already:** Surveys are increasingly implemented using electronic data collection. Adaptations to local context and translation into local language are important for successful data collection. Few published papers have examined the process and learning from implementing electronic data collection in large, multi-site surveys.	
**• What was done:** The EN-INDEPTH study surveyed 69,176 women with a household questionnaire, including randomised sections, undertaken in five sites, including rural population with limited internet availability. We undertook descriptive analyses and focus group discussions regarding field and office-based staff’s experiences to synthesise learning regarding design and implementation of electronic data collection, with implications for others doing large-scale population-based surveys.	
**WHAT DID WE LEARN?**	
**• Step 1—Selecting an electronic data collection platform:** data collection platforms differ and have very variable costs. We selected Survey Solutions since it was free, user friendly to programme, enabled each site to control their own interviews and data and enabled paradata collection.	
• **Step 2—Adapting and programming the questionnaire:** Our standard questionnaire was designed centrally with site-specific adaptations and translation into local languages. Site-specific piloting was important for successful data collection. It was helpful to have one site start first, as later sites could benefit from the cumulative experiences.	
**• Step 3—Selection and training of data collectors:** Standardised training materials were implemented. The training was found useful. Training shorter than 10 days was perceived to be too short. Training content and time should be adapted to the level of experience of data collectors.	
**• Step 4—Data collection process and data quality:** These were perceived to be facilitated by the electronic data collection platform, but challenges with synchronisation and GPS tracking were reported. Some sites used inbuilt dashboard to track data collection progress; however, across all sites, external tracking of data collection progress was implemented to allow for more detail.	
• **Step 5—Data management and analyses:** Data cleaning was perceived to be easier with fewer implausible values. Paradata was considered valuable to enable analyses of questionnaire timing and examining correction patterns.	
**WHAT NEXT IN MEASUREMENT AND RESEARCH?**	
**• Measurement improvement now:**	
o Further strategies for managements of multiple interviews on tablets would be valued, as would innovative solutions for synchronisation and GPS tracking especially in settings with limited internet.	
o Improving the tools and the electronic platforms alone is not enough. The capacity of users, training, supervision systems and quality of interviews are all key to ensure optimisation of data.	
**• Research needed:**	
o More rigorous comparisons of paper-based versus electronic data collection for surveys may no longer be the most important question; instead, survey implementers need more rigorous comparisons between the multiple software platforms available.	

## Background

More than two-thirds of the world’s births are in countries where household surveys are the main data source for health outcomes and coverage of care. The most widely used nationally representative surveys are Demographic and Health Surveys (DHS), usually every 5 years including more than 60 countries [1]. United Nations Children’s Fund’s (UNICEF) Multiple Indicator Cluster Surveys (MICS) are another major multi-country survey platform.

Survey data collection using traditional methods typically includes several consecutive steps, among these are: (1) data collection using paper questionnaires, (2) data entry and (3) data cleaning. Electronic data collection systems have enabled combining these three steps, making the data collection process more flexible and allowing for immediate verification of inconsistencies and reducing missing data [2–4]. A recent randomised comparison found more data errors when using traditional data collection compared with electronic data collection and that the errors were not randomly distributed [5]. Thus, electronically collected data may be more reliable and less biased.

Until recently, most household surveys have used a traditional pen-and-paper personal interviewing method for data collection. However, in the last decades, technological advances have allowed for new electronic data collection applications, and within recent years, there has been a rapid move towards electronic data collection [2, 6]. However, few published papers have reported on the process of using electronic data capture in large-scale surveys and learning from this process.

The Every Newborn-International Network for the Demographic Evaluation of Populations and their Health (EN-INDEPTH) study was a cross-sectional multi-site study aiming to inform improvements in measurement of pregnancy outcomes in population-based household surveys [7, 8]. The primary objective of the study was to compare two methods of retrospective recording of pregnancy outcomes: full birth history with additional questions on pregnancy losses (FBH+), as per the current standard in the seventh wave of Demographic and Health Surveys (DHS-7), and a full pregnancy history (FPH). The study also investigated the performance of existing or modified survey questions regarding other pregnancy-related outcomes and undertook qualitative research regarding barriers and enablers to reporting of these outcomes. Details of the study protocol and results of the randomised comparison have been published elsewhere [7, 8].

In this paper, we describe the process of identifying, implementing and using electronic data collection in the EN-INDEPTH study; we also provide qualitative research results on the field and office-based staff’s and site representatives’ perceptions on the challenges with electronic data collection.

## Methods

### Study setting

The EN-INDEPTH study was undertaken in five INDEPTH health and demographic surveillance system (HDSS) sites: Bandim in Guinea-Bissau, Dabat in Ethiopia, IgangaMayuge in Uganda, Matlab in Bangladesh and Kintampo in Ghana, supported by a joint team from Makerere University School of Public Health (MakSPH) and the London School of Hygiene & Tropical Medicine (LSHTM). The EN-INDEPTH study was undertaken between July 2017 and August 2018 including a total of 69,176 women of reproductive age. As a significant scope of the EN-INDEPTH study was to inform DHS survey improvements, members of the DHS were part of the expert advisory group (EAG).

### Description of processes

We described the steps of implementing electronic data collection in the EN-INDEPTH study, including identifying and adapting the electronic data collection platform, training of data collectors, management of interviews and monitoring of survey progress. Both information from the HDSS site representatives, MakSPH and LSHTM team members, were included.

### Qualitative methods

Six focus group discussions (FGDs) were conducted between February and May 2019 to describe the experiences with training, implementing and monitoring data collection using the Survey Solutions® data collection platform. Participants were mainly field- or office-based staff who used the Survey Solutions® application to either collect data in the field or manage data in the office in the EN-INDEPTH survey (Table 1). An FGD guide was developed and piloted with project managers, site coordinators, MakSPH and LSHTM team members during the EN-INDEPTH analysis workshop in February 2019 in Entebbe, Uganda. The adapted FGD guide was distributed in English to all site representatives (Additional file 1). The interviews were conducted and transcribed in English and local languages where applicable with translation to English.

**Table 1:** Characteristics of focus group discussion interviewees for the five sites, EN-INDEPTH study

	All sites and LSHTM team	Bandim	Dabat	IgangaMayuge	Kintampo	Matlab
Number of FGD interviewees	9	8	6	7	11	5
**Sex**						
Males	7	2	6	4	3	3
Females	2	6	0	6	8	2
**Role in the EN-INDEPTH study**						
Data collectors	0	8	4	7	7	0
Supervisors	1	0	0	2	0	4
Site coordinators	3	0	1	0	3	1
Site PIs	3	0	1	0	1	0
LSHTM/MakSPH team members	2	0	0	0	0	0

Thematic analyses were undertaken in NVivo version 12 using an iterative process guided by a priori themes developed from the interview guide. The guide was developed based on expert opinion and from the objectives of the study. New themes were included as they emerged during the analysis.

## Results

### Step 1: selecting an electronic data collection platform for a population-based survey

During the planning stage of the EN-INDEPTH study (late 2016–early 2017), an assessment of the previous data collection experience of the five HDSS sites was undertaken to inform the choice of data collection hardware and software. Sites were asked for information about their routine data collection practices, previous experience with data collection software and hardware and the technical capacity at the sites to examine the feasibility of data collection using either local or virtual servers hosted by LSHTM (Additional file 2). This assessment showed that most sites had some previous experience with electronic data collection using the Open Data Kit software, and had previously used Android OS-operated tablets, with some sites being well-equipped to use their own servers for data collection and storage.

In March 2017, a review of available electronic data collection software and platforms was conducted. Potential platforms were identified by reviewing electronic platforms used in major household surveys such as DHS and MICS and conducting an internet search. Platforms were reviewed using a list of prerequisites for the data collection tool, based on the requirements for making the electronic data collection process equivalent to the traditional paper-based data collection (to mimic the DHS data collection process), cost and other technical aspects (Additional file 3).

Accounting for sites’ previous experience, existing technical capacity (i.e. tablets and servers) and substantially higher costs associated with iOS-based tablets, the review focused only on Windows OS- and Android OS-based tablet applications to be used for the EN-INDEPTH study. Five eligible data collection applications and platforms were identified: CSPro (Windows OS version), CSPro (Android OS version), Qualtrics, Open Data Kit and Survey Solutions® (Table 2). The review results were shared with the EN-INDEPTH study EAG members for feedback, and a final choice of the data collection platform was made.

**Table 2:** Summary of electronic data collection platforms considered for use in EN-INDEPTH study

Platform/software	Site’s tablet availability and software use experience	Implications for use in EN-INDEPTH	Conclusion based on study pre-requisites
**CSPro, Windows OS version**	• No Windows OS-based tablets available at site • None of the sites have had experience using CSPro (Windows OS version) data collection platform	• No licence purchasing required (free) • Used for data collection at 75–80% of all DHS countries • Allows to display complex tables, i.e. birth history rosters, on one screen • Mimics paper version of the questionnaire • Template for DHS VII available via collaborators • Requires Windows OS-based tablets that are more expensive than average Android-based tablet • Adaptation of the questionnaire for study requires strong coding skills and knowledge of ASCII language • Limited user support	• Major investment required in each site for hardware, software and training, not useful for sites afterwards • Major set up and annual running costs—not sustainable for the sites
**CSPro, Android OS version**	• Limited number of Android OS-based tablets available • None of the sites have had experience using CSPro (Android OS version) data collection platform	• No licence purchasing required (free) • Template for DHS VII available via collaborators • Displays one question on a page • Does not support creation and displaying complex tables (i.e. rosters) • Adaptation of the questionnaire for study requires strong coding skills and knowledge of ASCII language • Limited user support	• Not functional for the birth-history questions required for EN INDEPTH survey • Would require in-house ASCII^1^ programmer (not available)
**Qualtrics data collection and management** **platform**	• Limited number of Android OS-based tablets available • None of the sites have had experience using Qualtrics platform	• Multi-site licence costs start from ca. $5000/year • The software allows to display the birth history/fertility history questions in one table • Adaptation is straightforward • The basic package includes Research Suite (the survey builder) • Template for DHS VII available via collaborators • User support available	• Major set up and annual running costs—not sustainable for the sites
**Open Data Kit (ODK)**	• Limited number of Android OS-based tablets available • Several EN-INDEPTH sites have some or limited experience using this platform for data collection • In-house ODK expertise available	• No licence purchasing required (free) • User support available, including user forum • Displays one question on a page • Does not support creation/displaying complex tables (i.e. rosters) • Adaptation of the questionnaire for study requires strong coding skills	• Questionnaire display not comparable to DHS questionnaires • Time consuming to programme • Not possible with per site headquarter/firewall
**Survey Solutions data collection and management** **platform**	• Limited number of Android OS-based tablets available • None of the sites have had experience using Survey Solutions data collection and management platform	• No licence purchasing required (free) • Design of the questionnaire is done online in Designer • Possibility to test questionnaire using Tester app • Summary of data can be displayed (mimicking roster) using limited and restricted HTML code • Possibility to link questions to specific household members listed in the roster file • Administration of data collection via system’s “Headquarter” with integrated dashboards, survey administration, data exporting, monitoring and reporting tools • Live monitoring of data collection, including GPS maps^2^ and accept/rejection • Extensive online technical support available, including active user forum, and online video tutorials and manuals • Possibility to collect GPS data and survey para data • By default does not support creation and displaying complex tables (i.e. rosters) • No template for DHS VII available • Minimal technical and programming skills required for survey adaptation and survey coding	• Preferred solution by sites, LSHTM, and agreed by DHS experts as the best option for this study

^1^American Standard Code for Information Interchange—coding language

^2^Upcoming planned feature in 2017, rolled out shortly after

Note: iOS-based applications were not considered as an option due to higher retail and repair price for iOS-based devices compared with Android OS-based devices

The World Bank’s Survey Solutions® electronic data collection and management platform (hereafter Survey Solutions) [9] was identified as the preferred data collection system and platform since it fulfilled most of our selection criteria: (1) minimal technical and programming skills required for survey adaptation and questionnaire coding; (2) relatively low technical requirements for Android OS-based tablets and servers; (3) no user fees; (4) existing online video tutorials, manuals, active user forum, and extensive online technical support; (5) possibility to link questions to specific household members listed in the roster file and displaying several questions on one screen (Table 2).

### Step 2: adaptation and programming of the questionnaire

The Microsoft Excel version of the DHS-7 module women’s questionnaire and the reproductive module of the Nepal 2016 women’s questionnaire were adapted to meet the EN-INDEPTH study objective [7].

The questionnaire was coded using the online Survey Solutions’ Designer web portal by a data analyst with no previous experience in Survey Solutions or in coding electronic questionnaires, with additional technical guidance from the World Bank Survey Solutions technical support team. The abridged demo application version was presented and tested during a multi-site design-and-implementation workshop in April 2017 in Dhaka, Bangladesh. Data storage, sharing and protection were discussed, and two sites (Matlab and Kintampo) decided to use their local servers for data collection, whilst three sites (Bandim, Dabat and IgangaMayuge) opted to use the virtual servers hosted at LSHTM (Table 3). Please see additional material for tablet and server requirements (Additional file 4).

**Table 3:** Site-specific context regarding electronic data capture experience and server setup, EN-INDEPTH study

	Bandim	Dabat	IgangaMayuge	Kintampo	Matlab
**Previous experience with electronic data collection**	Qualtrics	Open Data Kit and Open Health and Demographics System	Open Data Kit	Household Registration System 2 (HRS2)	SQLite
**Server hosting for EN-INDEPTH study**	LSHTM	LSHTM	LSHTM	Local	Local

**Fig. 1:**
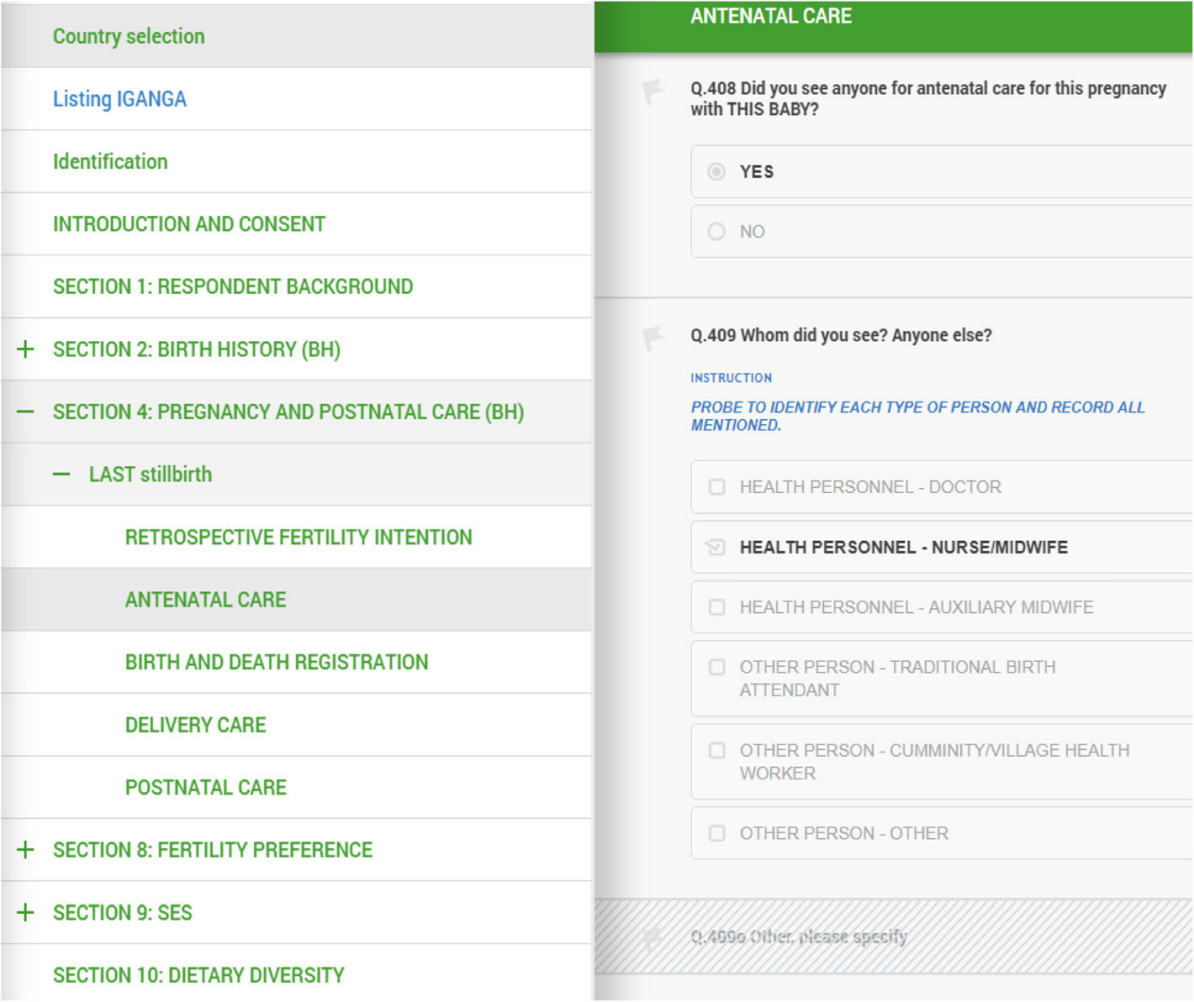
Screenshots from Survey Solutions application, EN-INDEPTH study

The questionnaire and application’s content and functionality (Fig. 1) were piloted in the Bandim site in July 2017. As this was the first site to implement the survey, several revisions of the coding of skip patterns, error prompts and notifications were conducted. Initiation of the data collection was halted after the pilot period to implement further changes. All changes were implemented before initiation of data collection at the other sites, enabling shorter pilot phase in other sites. Site-specific adaptations (i.e. translation into the local language, using context-appropriate wording, inclusion or exclusion of particular questions and/or sections) were made at all sites (Table 3). To minimise any possible disruptive impact of updates on the ongoing data collection, a single update with minor corrections to the content of the data collection app was conducted in January 2018 alongside the server software update.

#### Our experiences with adaptation and programming of the questionnaire

The process of adapting the questionnaire using the web-based Survey Solutions’ Designer was reasonably straight-forward and required minimal programming skills. The Survey Solutions’ Designer allowed for adding various question formats (e.g. single- or multi-select categorical, numeric, date, text, lists, GPS, picture) and specify their variable names, labels and pattern for alpha-numeric values, sub-sections, roster, static text and variables (computable expression). It furthermore allowed for coding which questions should be displayed based on specified conditions and answers to other questions. The adaptation from Excel version of the questionnaire required coding of the reverse logic of the skipping patterns between questions by programming related enabling conditions. We found the feature that enabled uploading of the country-specific translation of the questionnaire and generation of the resulting questionnaire in a pdf format particularly useful.

Most of the technical questions related to coding could be swiftly resolved using the existing video tutorials, the information provided at the support portal, user forum and helpful Survey Solutions technical support team. For text questions and static-texts, we found the ability to format text using a series of basic HTML tags particularly useful (e.g. for text bolding, underlining and colour-coding). At the time of the survey, the platform did not directly support presenting rosters as a summary table, required for our study. This was overcome by using a combination of the HTML tags and variables with guidance from the Survey Solutions technical support team.

### Step 3: selection and training of data collectors

Data collectors were recruited through interviews or among existing HDSS staff. Most sites mainly recruited data collectors not working with the HDSS data. Most of the selected data collectors had previous experience with DHS surveys (ranging from 0% in Matlab to 95% in IgangaMayuge) or other surveys (ranging from 42% in Dabat to 80% in IgangaMayuge) (Table 4). In three sites, most data collectors were female, whereas in IgangaMayuge 50% were female and in Kintampo 14% were female. All data collectors who received training were used for the EN-INDEPTH study. None of the data collectors had previous experience with Survey Solutions.

**Table 4:** Selection and training of data collectors, EN-INDEPTH study

	Bandim	Dabat	IgangaMayuge	Kintampo	Matlab
**Data collectors and supervisors**					
No. of data collectors	14	40	20	22	20
No. of supervisors	2	6	3	2	11
**Data collector characteristics**					
Male	2 (14%)	3 (7%)	10 (50%)	19 (86%)	0
Female	12 (86%)	37 (93%)	10 (50%)	3 (14%)	20 (100%)
Experience with DHS surveys	6 (43%)	33 (83%)	19 (95%)	13 (59%)	0 (0%)
Experience with other non-DHS surveys	7 (50%)	7 (18%)	16 (80%)	16 (73%)	14 (70%)
Have children	9 (64%)	7 (18%)	19 (95%)	13 (59%)	13 (65%)
**Training**					
Pre-training	No	No	Yes—consent form and study manual for self-study	Yes—intro session to explain difference between HDSS system and survey	No
Training with paper-based questionnaire	5 days	5 days	5 days	5 days	3 days
Training with Tester App	3 days	5 days	3 days	3 days	1.5 days
Total training time	8 days	10 days	8 days	8 days	4.5 days
Piloting in the field^1^	11 days	5 days	2 days	2 days	2 days
LSHTM/MakSPH team member(s) participation	Yes	Yes	Yes	Yes	No

^1^The longer pilot period at Bandim site was because Bandim was the first site to implement the survey, and the pilot covered pilot of both app and content. Section 4 of the survey (on pregnancy and postnatal care) was reduced significantly during the pilot, and many other issues raised during the pilot at the Bandim site were relevant for all sites

The EN-INDEPTH site teams with LSHTM and MakSPH teams jointly developed a standard training manual [10] on the data collection procedures, adapting the standard DHS Interviewer’s manual [11]. The manual was translated into local language and its content tailored to the specific country context and the HDSS site.

The training of data collectors and supervisors was led by the HDSS team with initial support from the LSHTM or MakSPH team in all HDSS sites, except in Matlab. Training at all sites started with the paper-based questionnaire (with emphasis on content), followed by tablet-care-and-use training, hands-on data collection exercise using the Survey Solutions Tester application, a classroom-based mock data collection training session using Survey Solutions’ Interviewer application, followed by field practice. Training duration ranged from 4.5 days in Matlab to 10 days in Dabat (Table 4), with the length of training shorter if data collectors were already familiar with tablet use and survey forms. After the training, all sites initiated the pilot phase of data collection. During the data collection phase, interviewing skills were maintained through supervision of data collectors. In the Dabat site, rapid turnover of data collectors required continuous training sessions.

Prior to initiation of the data collection, additional training on survey management using Survey Solutions’ online headquarters was provided to supervisors and data analysts on sites. To support these trainings, four additional manuals (data collection setup, Survey Solutions data management procedures, listing process, Survey Solutions Tester/Interviewer application) were developed adapting existing World Bank’s Survey Solutions Manuals [12]. A regular communication channel via e-mails was set up to resolve any technical and practical issues.

#### Respondents’ perspectives on training

Many respondents mentioned that the training had insufficient time especially for tablet practice. Others mentioned that the number of days needed for training is dependent on the level of experience of the trainees as well as the volume of questionnaires on the training application. Adaptations to local context, correction of skip patterns and translation were perceived to slow the training, although it was essential for data collection.

*I will recommend more days, maybe 3 weeks. It seems some of us were not used to the tablet, so if more days were added we would have gotten used to it (Site FGD, Kintampo, Ghana).*

*Days were not enough partly due to translating of the consent form and there were issues with logistics (Site FGD, IgangaMayuge, Uganda).*

Most respondents found the training interactive with brainstorming and recap sessions. The aspects of the training that were most appreciated included the screen projections of training contents, the theoretical aspect of the training, variations in the training methods and that each trainee had their own tablet.

*Yes, it was very much interactive, the group work and role plays made it very interactive and interesting and because it was the first time some of us were going to use tablets to collect data, we were very excited (Site FGD, Kintampo, Ghana).*

Suggested modifications to training included bulkiness of training materials in IgangaMayuge. Some data collectors suggested skipping training on paper-based questionnaires; however, most site representatives found the paper-based training useful for content focus. Internet connectivity was a problem for training particularly in the African sites.

*Would have been good to have tablet from the start instead of paper forms. To save time and learn quicker (Site FGD, Bandim, Guinea-Bissau).*

### Step 4: data collection, management of interviews and monitoring of survey progress and data quality

#### Allocation and management of interviews and data collection

**Fig. 2:**
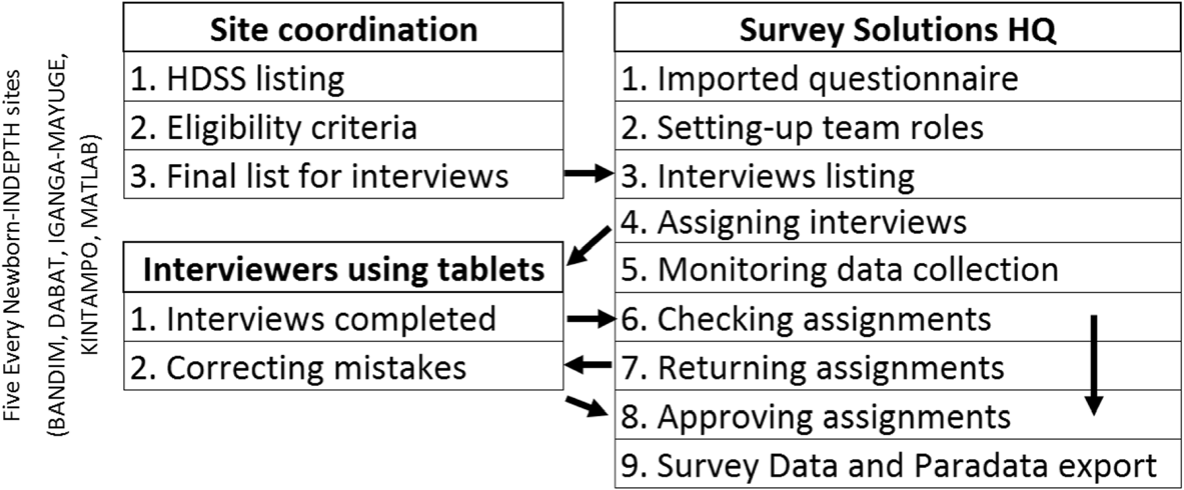
Diagram of data collection process, EN-INDEPTH study

In each site, a list of eligible women was extracted from the HDSS database. Based on this list, interviews were assigned to a supervisor, who assigned the interviews to the data collectors (Fig. 2). In most sites, data collectors were assigned a smaller number of interviews every day, whereas, in Bandim and Matlab, the data collectors were assigned a bulk of interviews before travelling to rural areas (Table 5). In most sites, the number of interviews assigned per bulk was increased as the data collectors gained experience.

**Table 5:** Management and monitoring of interviews in the EN-INDEPTH study

	Bandim	Dabat	IgangaMayuge	Kintampo	Matlab
**GPS location**^**1**^	No	No	Yes	Yes	No
**Assigning interviews**					
No of interviews assigned per interviewer per synchronisation	10 to 150	3 to 10	10 to 20	20 to 25	60 to 150
Responsible for assigning interviews	Site representative and supervisors	Field coordinator	Supervisors	Supervisors	Supervisors
**Monitoring**					
Survey Solution Headquarters progress	No	No	Yes	Yes	No
Separate tracking of progress^2^	Yes	Yes	Yes	Yes	Yes
**Data cleaning**					
Separate system	Yes	Yes	Yes	Yes	Yes

^1^The GPS function was enabled at all sites at the start of the survey. Sites with “Yes” maintained the GPS function during the survey. Sites with “No” disabled the GPS function

^2^Using Stata®, a do-file was created for sites to track the progress of the data collection to report progress every 2 weeks to the LSHTM team

The length of the data collection varied by site depending on the fieldwork schedule and allocated sample size, ranging between 6 and 13 months (Additional file 6). In all sites, data collectors tried to locate the women to be interviewed through household visits (three attempts). Data collection was performed offline. At the end of the day (or end of interview round), completed interviews were synchronised and uploaded to the designated server. Interviews that were not successfully completed were either re-visited or uploaded to Survey Solutions Headquarters and reassigned to another interviewer (Fig. 2).

#### Respondents’ perspectives on allocation of interviews and data collection

Survey Solutions was perceived to facilitate data collection through skip patterns and error messages. Initially, error messages were not always correct and relevant. This was corrected in collaboration between field assistants, site representatives and the LSHTM team.

*It also prevents us from mistakes by reminding us when we wrongly try to enter data for example: If the first question “Have you eaten your lunch?” The response is “No” and for the next question “what type of meal did you eat?” The tablet prevents you from filling this by saying that “you didn’t eat at all!” (Site FGD, Dabat, Ethiopia).*

Slow synchronisation due to slow internet connection was a challenge at all sites. More sites reported challenges with the tablet freezing or closing down leading to loss of data necessitating re-interview of some women (this issue was fixed with a later software update).

*The largest disadvantage was loss of data if the app closed down – data was lost for both what they are working with and other interviews not synchronised. Therefore, they had to be repeated, which was not always very welcome both by women and data collectors (Bandim member, Entebbe workshop, Uganda).*

*During the interview time the tablets would all of a sudden hang (get stuck) – so the interview stopped and restart the tablet (Matlab member, Entebbe workshop, Uganda).*

The GPS tracking feature was disabled in some sites, as it was battery-draining and time-consuming with the delayed signal search. Sites experienced more problems with the GPS feature during the rainy season. Kintampo moved the GPS feature to the end of the questionnaire in order not to halt the interview process.

*A negative aspect was on how to synchronise assignments. Most of our villages were not having network…You would move around the compound and take a long time before you could pick GPS coordinates (Site FGD, Kintampo, Ghana).*

As it was essential for data collection that the battery of the tablet could last for at least a full working day, most sites found it necessary to buy power banks.

*We had to buy extra power banks to give enough support. On average 5 to 6 interviews per day, went up to 7-8 at the end (Matlab member, Entebbe workshop, Uganda).*

Data collectors from Bandim adapted separate paper-based lists to keep track of the number and order of children. This was necessary because it was difficult to make corrections to the roster on the tablet.

*We used paper to register all children and the order of the children first and then afterwards entered the information on the tablet, in order to calculate and not loose information (Site FGD, Bandim, Guinea-Bissau).*

Finding the correct interview on the tablet was a challenge where a bulk assignment of interviews was used. In Matlab, IgangaMayuge and rural Bandim, parallel paper-based lists with information about women to interview were used to keep track of the number of interviews and notes for women not identified because of the difficulties in managing large number of incomplete interviews on the tablet. A reliable search function could facilitate finding the correct interview.

*At first we did not have lists on paper in the rural area – and that was difficult. Some times in the rural area, it was hard to distinguish between two regions because this was not displayed on the tablet. It was hard to do it by hand (Site FGD, Bandim, Guinea-Bissau).*

Several challenges around effectively assigning and re-assigning interviews were noted partly due to poor internet connection, but sites also mentioned the lack of being able to order and select multiple interviews based on a set of criteria, when reassigning interviews. This was particularly an issue towards the end of data collection when remaining interviews were dispersed.

*Internet was too slow and synchronising took long and some did not get the questionnaire. So it was a big problem (Site FGD, IgangaMayuge, Uganda).*

*I want to talk about the negative experiences of reassigning work to supervisors… the women could not be located and supervisors or a different interviewer was to look up for those women, it leads to a situation whereby assignment were scattered across, so one field worker could have assignments in like three different communities... (Site FGD, Kintampo, Ghana).*

#### Monitoring of survey progress and data quality

**Fig. 3:**
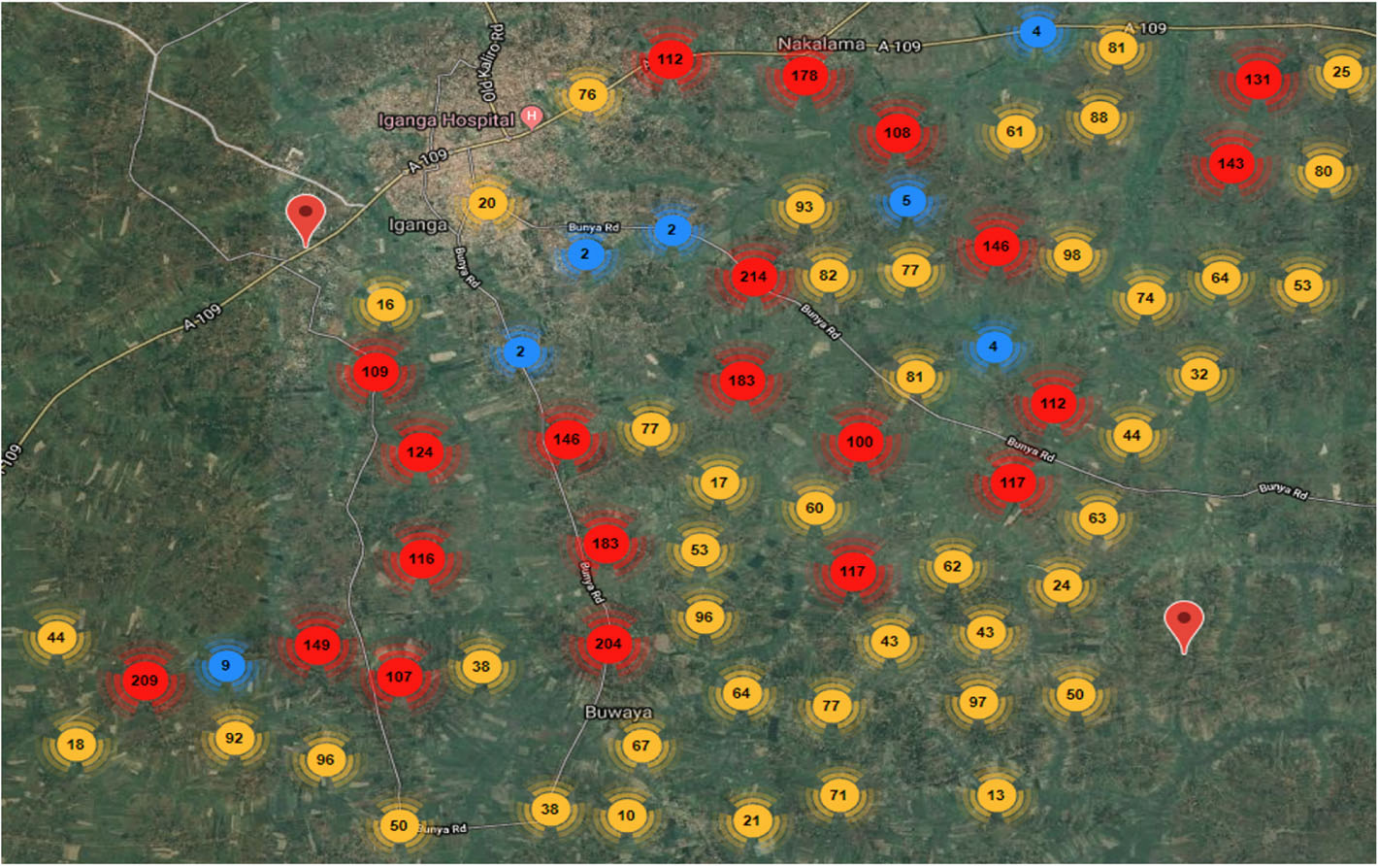
Map of GPS location of data collectors in a sample site (IgangaMayuge), EN-INDEPTH study

**Fig. 4:**
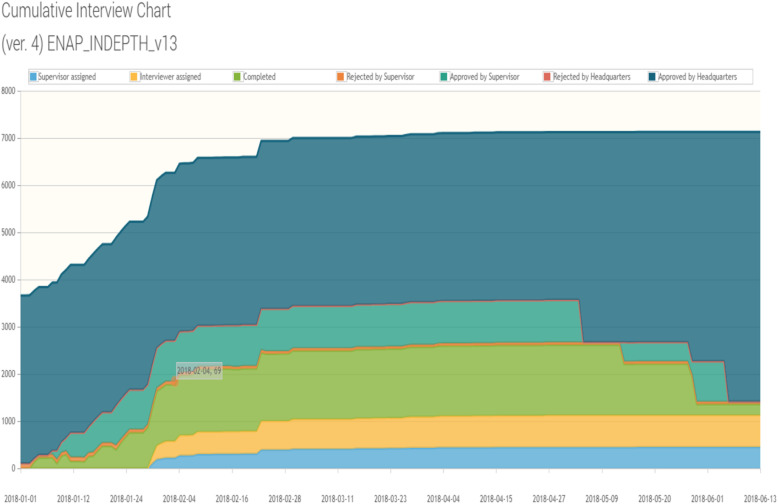
Screenshot from Survey Solutions Headquarters’ tracking of interviews, EN-INDEPTH study

The Survey Solutions Headquarter displayed the progress of data collection, including interview duration, speed and GPS coordinates (Fig. 3), as well as additional data (paradata) (Headquarters tracking in Fig. 4). The paradata contained information on the data collection process including timestamp data for the beginning and end of question section and corrections made. We used these data to examine the feasibility of its use to enhance household pregnancy questionnaire design and survey implementation [13]. In the Survey Solutions’ Headquarters, it was possible to review the interview content and send it back, when necessary, leaving comments (screenshot examples in Additional file 5). To ensure that potential errors were checked, all sites except Matlab, used a do-file generated in Stata 15® to identify inconsistencies between the number of children in the roster and the summary birth history (for both FBH+ and FPH interviews), as this would likely be overlooked using only the Survey Solutions’ Headquarters.

The Survey Solutions’ Headquarters allowed to follow the progress of the number of interviews completed and approved. Aside from the tracking of interviews in the Survey Solutions’ Headquarters, every 2 weeks, the progress of interviews was also monitored for each site by extracting data and analysing the progress using Excel monitoring tables (Additional file 7) generated using Stata 15®.

#### Respondents’ perspectives on monitoring of survey progress and data quality

The Survey Solutions’ Headquarters was reported as useful by office staff in monitoring progress of work (tracking number of interviews completed per interviewer), data quality monitoring (reviewing and rejecting or accepting interviews). Some sites reported the lack of choosing criteria to be checked in addition to reviewing the interviews individually, as the latter might lead to some important inconsistencies being overlooked. GPS coordinates made it possible to see the particular place the interview was conducted (Fig. 3).

*We were using the dashboards on the Survey Solutions’ Headquarters, so there are dashboards that tell you the number of interviews a fieldworker has done and the number of rejected or accepted interviews, so we can access the performance of field worker right here (Site FGD, Kintampo, Ghana).*

In addition to the quality checks performed based on the data collected, data collectors were supervised during the data collection to ensure that the quality of the interviews remained adequate during the time of the study.

*There was a Field Supervisor who would do sit-in-interviews to oversee the entire process and after doing it for a number of times to a particular person, she went to another person (Site FGD, IgangaMayuge, Uganda).*

### Step 5: data management and analyses

Interview data were entered on Android-based tablets using the Survey Solutions platform. Data and paradata (interview duration, speed, GPS coordinates and additional data) were stored locally on the tablets and synchronised regularly whenever internet was available [7]. Following synchronisation, data from tablets were uploaded to the country’s dedicated virtual or physical server with regular automatic back-up and additional back-up on a separate server or external hard drive. Survey Solutions allowed for different user roles with varying level of permissions and functions: interviewer (data collection), supervisor (assigning and monitoring data collection by data collectors), headquarters (overall survey and data management) and observer (monitoring, not used in this study). Raw data were stored in an encrypted format, accessed only by the country’s data manager. Anonymisation of the quantitative and qualitative data took place in-country before data sets were pooled into one multi-site data set for analyses.

Survey Solutions’ Headquarters allowed for exporting of the main survey data and paradata in several formats (Stata®, SPSS® or tabular). Exporting of such data was also possible per version of the survey questionnaire, as well as by status of the interviews (interviewer assigned, completed, approved by supervisor or by headquarters). The Survey Solutions platform does not contain any database manipulation tools; however, it includes a flexible application programme interface, which allows automating some tasks (e.g. external dashboards, checking and validation or data export). In our study, we opted not to utilise this functionality.

#### Our experiences with data management and data analyses

In general, we found that data cleaning and validation was overall easier (compared with our previous experiences) owing to the presence of fewer implausible values specified by validation conditions and error or warning messages. The paradata collected during our survey was used to analyse the timing of data collection and examining the answer correction patterns [13]. Data management and analyses using paradata was challenging because of very large file size (~ 90 GB) and different data structure between different versions of Survey Solutions.

## Discussion

This paper synthesises learning from the design, set up and implementation of a large, population-based survey, covering 69,176 women in five countries, including hard-to-reach rural populations with limited internet access. Whilst there is a shift from paper to electronic data capture for large-scale surveys, few papers have described learning on what works (and does not) for large-scale surveys, and the steps and decisions involved. We have identified five points with decisions required and used qualitative data to understand these more.

Based on the prerequisites for this study, we selected the World Bank’s Survey Solutions as the preferred data collection platform for this particular study, importantly that the software allowed questionnaire design comparable to DHS use for the pregnancy roster, that there was not high cost for the site teams and that sites could own their own data. After review of the options with the teams, the final choice of software was made by the LSHTM team with insights from EAG members [8]. We specifically consulted EAG members, who were DHS staff, regarding the questionnaire design and layout discussions. Many of the platforms we reviewed have advanced considerably since, and our review should therefore be used mainly for illustrative purpose of deciding on the study or programme-specific pre-requisites when selecting between the many possible electronic data collection platforms. In line with others, we recommend that local context, design and aim of study; costs and experience of both questionnaire coder, data collectors and supervisors; and technical support options be considered when choosing electronic data collection platform [14]. Other studies have discussed transition from paper-based to electronic data collection in smaller studies [2, 15, 16].

The online technical support by the World Bank Team allowed that a data analyst without previous coding experience could code the questionnaire on a short time. Adaptation to local settings including translation to local languages was useful for successful data collection, although these initial adaptations slowed the training in some sites; this was perceived to be important for successful data collection.

The training was led by each HDSS team with support by the same LSHTM and MakSPH team members in all but one site. Standardised training materials enabled uniform training across sites, but length of training varied by sites. When implementing an electronic data collection platform in a multi-site study, it is worthwhile to consider standardising both training material and length of training; both should be adjusted to the level of experience of data collectors. It was useful to have one or more person(s) participate in training in most sites. Our learnings stress the importance of local adaptations and a local pilot study to ensure that most of the problems related to the content of the questionnaires, skip patterns and error prompts are resolved in time, and to assess whether GPS tracking options will work in the local setting. In multi-site studies, we recommend piloting the survey in one site prior to expanding the data collection to other sites as this allows for resolving most issues with skip patterns and error messages, and thus shorter pilot phases in other sites.

The consensus from data collectors and site teams was that electronic data collection (in this case Survey Solutions) had several benefits over traditional paper-based data collection. Notable advantages included smoother data collection process with benefits of skip patterns, error prompts and reminders if required fields were not answered. We did not compare paper-based and electronic data collection. However, our reported perceptions of higher data quality are consistent with a study from Nepal and a study from Malawi, where missing data were twice [17] and three times [15] as frequent in paper-based compared with electronic data collection. As in our study, data collectors from Malawi also preferred electronic data collection [15] whereas a randomised field experiment from Tanzania reported no difference in how smooth the interview process was perceived in paper-based and electronic data collection [5]. Data cleaning was perceived to be easier with fewer implausible values.

Potential to integrate GPS tracking is another advantage of electronic data collection systems. In this study, this was only implemented in two sites as poor GPS signal slowed down the GPS tracking. In one of the sites, GPS tracking was moved to the end of the questionnaire, in order not to halt the interview process. Similarly, in an Ethiopian trial, GPS tracking problems necessitated dropping GPS data collection for some interviews [18]. However, overall, these challenges are still likely lower than those faced when undertaking manual GPS readings in paper-based surveys according to a study reporting GPS problems in 6.6% of paper-based interviews and only in 0.6% using electronic data collection [5]. Issues like synchronisation and data transfer should be given special consideration when choosing an electronic data collection platform.

Recent guidelines on the use of electronic data collection in population surveys by United Nations Statistics Division recommend that data should be transferred to headquarters at least on a daily basis [19]. We also found this to be useful, but particularly in rural areas, this strategy is not always feasible. We, therefore, recommend that software developers and Survey Solutions in collaboration with users further develop organisation possibilities of multiple interviews (i.e. ordering and archiving) on devices and search functions, as these are crucial for the management of multiple interviews, and therefore important for data collection particularly in rural settings.

### Strengths and limitations

A strength is that this study draws from five geographically diverse sites in Africa and Asia. Our experiences are likely to be relevant across a variety of low-resource settings, as we included adaptations of DHS questionnaires, which are a common source of data collection in such settings. Following the completion of the study, two sites are in the process of integrating electronic data collection into their routine HDSS data collection; a further site member has used the experiences gained from this trial to conduct another survey using the Survey Solutions platform. As this was not a study planned from the inception of EN-INDEPTH, the descriptive data aspects around the choice of software, the adaptations and training included in this paper are based on notes, e-mail correspondence and memory. However, as this is a multi-site randomised comparison, study notes and e-mail correspondences were extensive.

Another strength of this paper is the use of a standard FGD interview guide across all sites with FGDs undertaken in local languages collecting the perspectives of both data collectors and assistants managing the interviews in the office. In the Matlab site, no data collectors participated in the FGD, but supervisors shared data collectors’ day-to-day problems, and these were reflected in the FGD. However, whilst this study presents a comprehensive overview of the perspectives of those involved in survey across the five sites and LSHTM and MakSPH teams, a gap is that no information was collected directly related to the women’s or communities’ experiences with the electronic data collection. Whilst there was extensive qualitative data collection from the women’s perspective, we focused on barriers and enablers to reporting pregnancies and adverse pregnancy outcomes. [20]. A study in Uganda found electronic data collection to be acceptable to women [21]. Future studies should consider the importance of including the respondents’ perspective. In particular, attention to digital health governance is important to protect vulnerable populations, including women in low-resource settings [22].

We did not undertake an economic evaluation regarding the electronic data collection process, which would add value in future research. We note that one other study in South Africa reported on experience of using mobile data collection [23], and the subsequent economic evaluation found that the mobile data system added significantly to running cost of the programme, although this may also reflect high internet data costs in South Africa [24, 25].

## Conclusions

Our experiences with electronic data collection in this multi-site household survey stress advantages and perceived value, but also the importance of considering many issues including cost and technical support when choosing an electronic data collection platform. Also, we provide recommendations for others considering using electronic data collection in multi-site surveys, i.e. to ensure adaptations to the local context, piloting the questionnaire in one site first, buying power banks to avoid power interruption and to give special attention to technical issues as GPS tracking and synchronisation, particularly in settings with poor internet.

## Supplementary information

**Additional file 1.** Interview guide for focus group discussions.

**Additional file 2.** Site’s data collection readiness assessment template.

**Additional file 3.** List of software requirements.

**Additional file 4.** List of tablet and server requirements.

**Additional file 5.** The World Bank Survey Solutions Headquarters overview.

**Additional file 6.** Overview of the ongoing routine and EN-INDEPTH study data collection at each site.

**Additional file 7.** EN-INDEPTH overall progress site report.

**Additional file 8.** Ethical approval of local Institutional Review Boards.

## Data Availability

Data sharing and transfer agreements were jointly developed and signed by all collaborating partners. The datasets generated during the current study are deposited online at https://doi.org/10.17037/DATA.00001556 with data access subject to approval by collaborating parties.

## Abbreviations

CSPro: Census and Survey Processing System
DHS: Demographic and Health Surveys
EAG: Expert Advisory Group
EN-INDEPTH: Every Newborn-International Network for the Demographic Evaluation of Populations and their Health
FBH+: Full birth history (+ denotes additional questions on pregnancy losses)
FGD(s): Focus group discussion(s)
FPH: Full pregnancy history
GPS: Global Positioning System
HDSS: Health and demographic surveillance system
ID: Identification number
LSHTM: London School of Hygiene & Tropical Medicine
ODK: Open Data Kit
MakSPH: Makerere University School of Public Health
