# Supplementary material for: Electronic data collection in a multi-site population-based survey: EN-INDEPTH study
Source: Popul Health Metr. 2021 Feb 8;19(Suppl 1):9. doi: 10.1186/s12963-020-00226-z (PMC7869201; doi:10.1186/s12963-020-00226-z)
Supplement: Supplementary file 1 — Additional file 1. Interview guide for focus group discussions. [file 12963_2020_226_MOESM1_ESM.docx]

# **Additional file 1: Interview guide for focus group discussions**

**FGD Guide on Survey Solutions data collection tool- Experiences from the field**

Aim: To share experiences from field/sites regarding the data collection tool and suggest ways to improve it for future use.

*Please explain to participants that the objective of this FGD is to share experiences regarding the data collection tool.*

Facilitator/moderator:

Notes taker:

The FGD will look at these themes/areas for discussion:

1. Training on the survey solutions software
2. Managing and assigning interviews in the office
3. Data collection period and process
4. Monitoring of the software on the field
5. Data analyses
6. Make recommendations for its improvement for future use

No right or wrong answer. We are here to share experiences.

**Background information from each site:**

| Name of site |  | Remarks |
| --- | --- | --- |
| Number of field workers trained for survey |  |  |
| Number of Supervisors trained for survey |  |  |
| Number of days for training (including pilot) |  |  |
| Number of staff involved in FGD |  |  |
| Date of interview for FGD |  |  |
| Name of moderator |  |  |
| Name of notes taker |  |  |
| Duration of interview |  |  |

**Part A: Training of staff on the survey solution data collection tool. Here we will discuss and share experiences on how the training for the survey solution went across sites**.

1. How was the training done? Who was involved in the training? Number of days for the training? Were the days okay or you would recommend for more days for the training?
2. Was the training interactive? Can you share some of your experiences from the training: What specific aspects did you like about the training? Was there anything you didn’t like about the training? What were they?
3. Were there any challenges with the training? How were the challenges overcome?

Do you have any suggestions on how the training could have been improved?

Were there any areas that you think were important that were not covered in the training?

What else would you like to have known before commencing data collection?

**Part B: Management of interviews in the office: Here we will share experiences from the office in the management of interviews (assigning and re-assigning), we will ask about your experiences from the office and how you overcame such difficulties**.

1. Can you tell me about how you assigned interviews to data collectors? Share both positive and negative experiences.
2. How were the challenges overcome?
3. If we are to do data collection again, what will be your recommendations to the office team? In your opinion, what will be the best way to manage interviews from the office using Survey solutions software?

**Part C: Data collection: Here we will share experiences from the data collection in general. In the site specific FGD with the fieldworkers, we will ask about their experiences from the field and how they overcame those challenges.**

Can you tell me about the experience of using Survey Solution app to collect data in the field?

1. Ask participants to share both positive and negative experiences.

How were the challenges overcome? Did you use any paper based notes or other aides to support your data collection with the app? If yes, how was it done?

1. If we are to do data collection again, what will be your recommendations to the field team? In your opinion, what will be the best way to collect data from the field using Survey solutions?

**Part D: Monitoring on the field-The aim here is to share experiences and document the field experiences**

1. How was the monitoring done? Describe the process
2. Did you use the Survey Solution options for tracking surveys per interviewer? Survey completion rates? See where interviewers were? Data quality?
3. Did you find these Survey Solution options useful or not?
4. Did you use other methods than the Survey solution options to monitor progress and quality?
5. Any recommendations for changes to the Survey Solution tracking options?
6. Any recommendations for future use?

**PART E: General recommendations for Survey solution data collection tool**

Would you recommend this tool to be made more widely available for field data collection? Why/Why not?

What do you think would be the most important thing to consider if this data collection tool is made widely available?

THANK YOU FOR YOUR TIME AND THE INFORMATION SHARED. DO YIU HAVE ANY QUESTIONS?

Moderator/Notes taker’s comments and observation:
