# Supplementary material for: Electronic data collection in a multi-site population-based survey: EN-INDEPTH study
Source: Popul Health Metr. 2021 Feb 8;19(Suppl 1):9. doi: 10.1186/s12963-020-00226-z (PMC7869201; doi:10.1186/s12963-020-00226-z)
Supplement: Supplementary file 2 — Additional file 2. Site’s data collection readiness assessment template. [file 12963_2020_226_MOESM2_ESM.docx]

# **Additional file 2: Site’s data collection readiness assessment template**

**Part 1 – Site’s data collection experience assessment**

1. When are you planning to start data collection? Please illustrate (using provided template) the relationship between the planned timeline of survey data collection and national/local schedules of HDSS data collection.
2. Are you ready to proceed with data collection (hardware)? Please confirm you have a sufficient number of tablets that will be used for data collection. If not, please indicate when do you anticipate having all the tablets necessary.
3. How the data will be collected (software)? Following your feedback in 2016, most have previously used ODK collect (<https://opendatakit.org/use/collect/>). To synchronize the data collection approaches across sites, we suggest using ODK as a platform for data collection. Please confirm that you agree to use ODK collect for data collection at your site. If not, please suggest an alternative solution that can be easily implemented across all sites.
4. How experienced is your team with ODK collect? Please indicate the level of experience/expertise using ODK collect; level of support you will require with ODK collect.
5. Do we agree to create the questionnaire for later uploading to ODK Collect centrally, with additional collaboration on creating the local translations, where necessary?
6. How will we randomise questionnaires? Do you have previous experience of running surveys with randomisation? If so, how did you manage implementation of randomisation logistically (app, excel, schedule, other)? Shall we manage randomisation locally or centrally?
7. How many data collectors are necessary for data collection? How many data collectors need a training in data collection, particularly using ODK collect? How many data collectors have sufficient experience to lead training of trainers’ activities?
8. Do you have experience of training data collectors? If you have previously developed training manuals for the user of the tablet and ODK collect software, we will be very grateful if you could please send a copy of these materials. We will then combine and make a standardised manual on ODK data collection to be used across all sites.
9. How experienced is your team with data management? Please indicate the level of support you will require with data management.
10. Who will lead on data collection? Please nominate (name and email) the key contact persons who will form the technical experts team for Skype calls related to data collection/management/app implementation.

**Part 2 – Site’s detailed data collection capacity and tablet readiness**

1. Number of data collectors:
2. Number of tablets already available for data collection (owned or can borrowed):
3. Number of tablets that will be bought (in addition to those specified above):
4. Brand and screen size (if known):
5. Platform and current version (ex. Android 6.0):
6. Name(s) and e-mail(s) of key Data Collection person(s):
7. Name(s) and e-mail(s) of key Data Manager(s):
8. Previous experience with Android app development (if yes, key person(s) who can be involved/consulted):
9. Software/apps used for data collection:
10. Software used for data management:

**Part 3 Site’s server readiness assessment**

1. Which server is currently being used or can be used at your site: cloud-based server (if cloud – which one?), physical server, none
2. Specification of the server that is currently being used or can be used at your site (CPU, RAM, free storage space):
   Example   -  4 cores CPU, 8GB RAM and 50GB free space
3. Operating system:
   Example   -  Windows Server 2012R2
4. Are the following software packages already installed:
   1. Microsoft IIS – yes/no (if NO, is it possible to install it?)
   2. Microsoft .Net Framework 4.6.1 – yes/no (if NO, is it possible to install it?)
   3. PostgreSQL (version 9.4 minimal) – yes/no (if NO, is it possible to install it?)
5. Is the server properly cooled? – yes/no
6. Is the server physically secured? – yes/no
7. Is the server reliably powered 24/7? – yes/no
8. Is any other survey (service) currently uses this server – yes/no (if YES, till when this service (survey) will use this server)
9. Do you use any specific firewall and/or antivirus or integrated security software? – yes/no (if YES, please specify)
   Example – McAfee Internet Security and ZoneAlarm ProFirewall
10. Are you able to provide Remote Desktop Protocol? – yes/no
