# Supplementary material for: Electronic data collection in a multi-site population-based survey: EN-INDEPTH study
Source: Popul Health Metr. 2021 Feb 8;19(Suppl 1):9. doi: 10.1186/s12963-020-00226-z (PMC7869201; doi:10.1186/s12963-020-00226-z)
Supplement: Supplementary file 3 — Additional file 3. List of software requirements. [file 12963_2020_226_MOESM3_ESM.docx]

# **Additional file 3: List of software requirements**

Main requirements for the platform:

- - Level of programming skills required for survey adaptation
  - Compatibility with Android- or OS-based tablets
  - Costs of the license
  - Availability of ongoing technical support
  - Previous experience in study sites
  - Feasibility of adaptions for use with EN-INDEPTH survey questionnaire including birth/pregnancy history
