# Supplementary material for: Electronic data collection in a multi-site population-based survey: EN-INDEPTH study
Source: Popul Health Metr. 2021 Feb 8;19(Suppl 1):9. doi: 10.1186/s12963-020-00226-z (PMC7869201; doi:10.1186/s12963-020-00226-z)
Supplement: Supplementary file 4 — Additional file 4. List of tablet and server requirements. [file 12963_2020_226_MOESM4_ESM.docx]

**Additional file 4: Tablet and server requirements**

**Tablet requirements:**

1. Tablets should be operating OS Android 5.0 or above. Devices running an older OS Android 4.1-4.4 are incompatible with the new security certificates used on servers.
2. 1GB of RAM and 8GB of flash memory storage are minimal. Better technical characteristics will improve responsiveness of the programme. A higher storage capacity is recommended.
3. At minimum 1GB of available space must be available for the Survey Solutions’ use. A higher storage capacity is recommended.
4. The tablets should be sim card enabled to allow for 3G or 4G connection for connectivity while in the field. Alternatively, you can use WIFI connection in the office to synchronize the data or use WIFI created by 3G/4G dongles.
5. The tablet should have cover for storage and protection from rain and sudden drops.
6. The tablet should have an extra external battery or recharger, particularly if you work in the remote area and do not have a stable supply of electricity.
7. It is recommended to install a tablet lock APP to limit the use of the tablets for other purposes by the interviewers other than data collection, as it can reduce the battery performance of the tablet up to 50%.
8. Please remember that the tablet will be assigned for use to one data collector only. It is not possible to use the tablet by two different people interchangeably. This is for security purposes. It is highly recommended to have spare tablets in case of tablets being lost or broken.

**Server requirements:**

1.       **Pre-installation phase server readiness check:**

1.1. Please check that **SERVER** meets the Physical prerequisites:

a.       4 cores CPU, 8GB RAM and 50GB free space as minimum requirements. Better hardware allows better performance. WB technical support recommended quadrupling the basic minimum requirements specified above (for RAM and drive), or having at least Intel Xeon E5-2670 v2 (Ivy Bridge) Processors,8GB RAM, 50-100 GB SSD Drive.

b.       Ideally, server is dedicated to the project and not competing with other projects for server resources.

c.       Internet access stable and is 24/7, fast and reliable.

d.       Server back-up is arranged (physical external or virtual drive), and regular data back-up is scheduled.

e.       It is possible to provide remote access (RDP) and admin rights to WB Survey Solutions tech team to install the software remotely (see 2.3 below ‘Application installation (step 2b)’ )

1.2. Please check that **SERVER ENVIRONMENT** meets the Server Environment prerequisites:

a.       Properly cooled

b.       Physically and digitally secured

c.       Reliably powered 24/7

d.       Technical support available

1.3   Please check that the **SERVER SOFTWARE** meet the Software prerequisites (installed and configured prior to Survey Solution installation):

a.       Windows Server 2012R2 or above

b.       Microsoft IIS (included in OS)

c.       Microsoft .Net Framework 4.6.1 or above

d.       PostgreSQL version 9.4 minimal, or above

1.4   Antivirus/firewall/integrated security package software installed. Firewalls, anti-virus, and integrated security packages need to be configured to permit Survey Solutions server component to communicate with other devices exchanging data with it.

a.       [Instructions for Windows Server 2012 Firewall](https://technet.microsoft.com/en-us/library/cc753558.aspx)

b.       [Avast Internet Security](https://www.avast.com/en-us/faq.php?article=AVKB25)

c.       [Comodo Internet Security](https://help.comodo.com/topic-72-1-451-4772-.html)

d.       [Kaspersky Internet Security](http://support.kaspersky.com/us/12101)

e.       [McAfee Internet Security](https://service.mcafee.com/webcenter/portal/cp/home/articleview?locale=en-US&articleId=TS100887)

f.        [Norton Internet Security](https://support.norton.com/sp/en/us/home/current/solutions/kb20100320035324EN_EndUserProfile_en_us)

g.       [ZoneAlarm Pro Firewall](http://download.zonealarm.com/bin/inclient/ZA_HelpCenter/91619.htm)
