# Supplementary material for: Electronic data collection in a multi-site population-based survey: EN-INDEPTH study
Source: Popul Health Metr. 2021 Feb 8;19(Suppl 1):9. doi: 10.1186/s12963-020-00226-z (PMC7869201; doi:10.1186/s12963-020-00226-z)

**Additional file 5: The World Bank Survey Solution Headquarters overview**

Sections overview: Reports, Interview, Teams and Roles, Survey Setup, Data Export, Help Settings


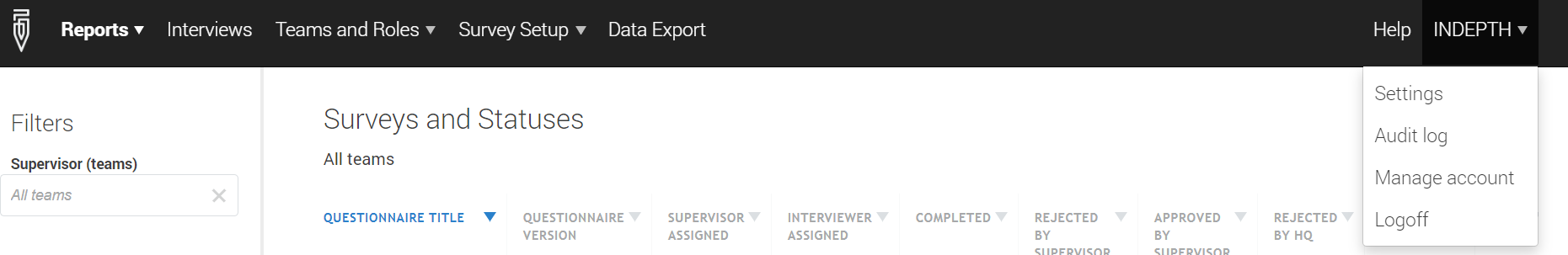


Reports - Surveys and Statuses


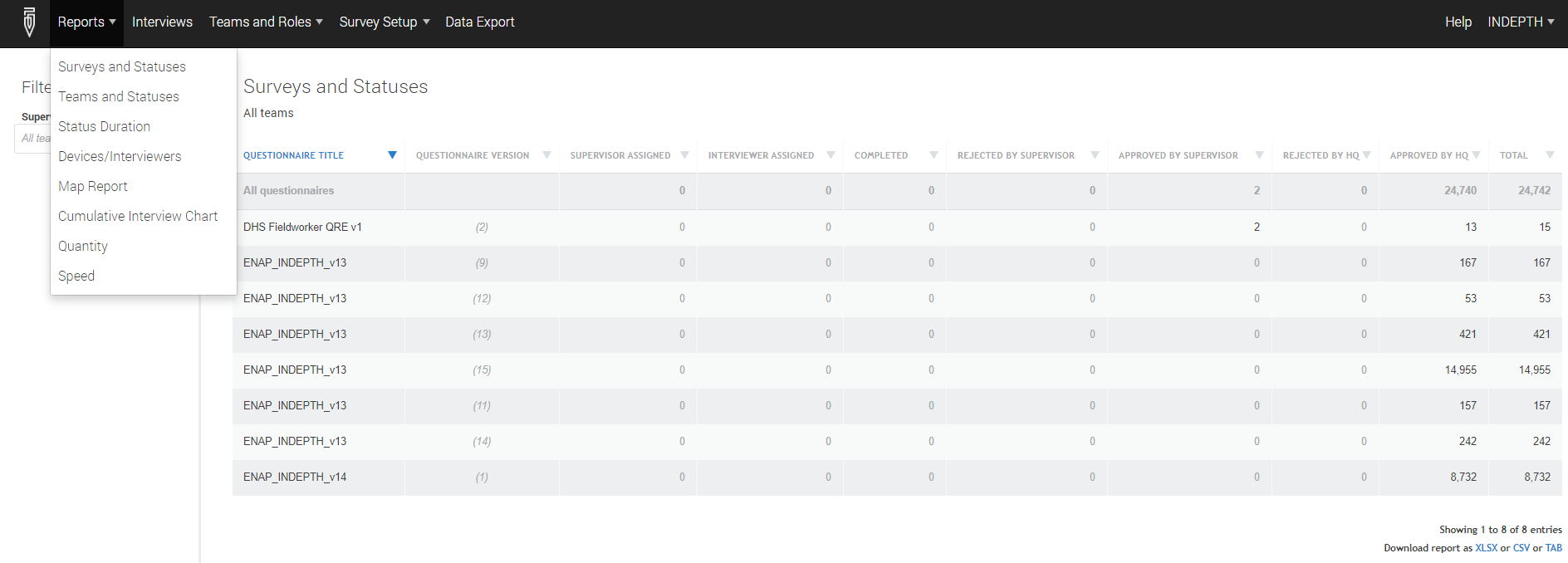


Reports – Teams and Statuses


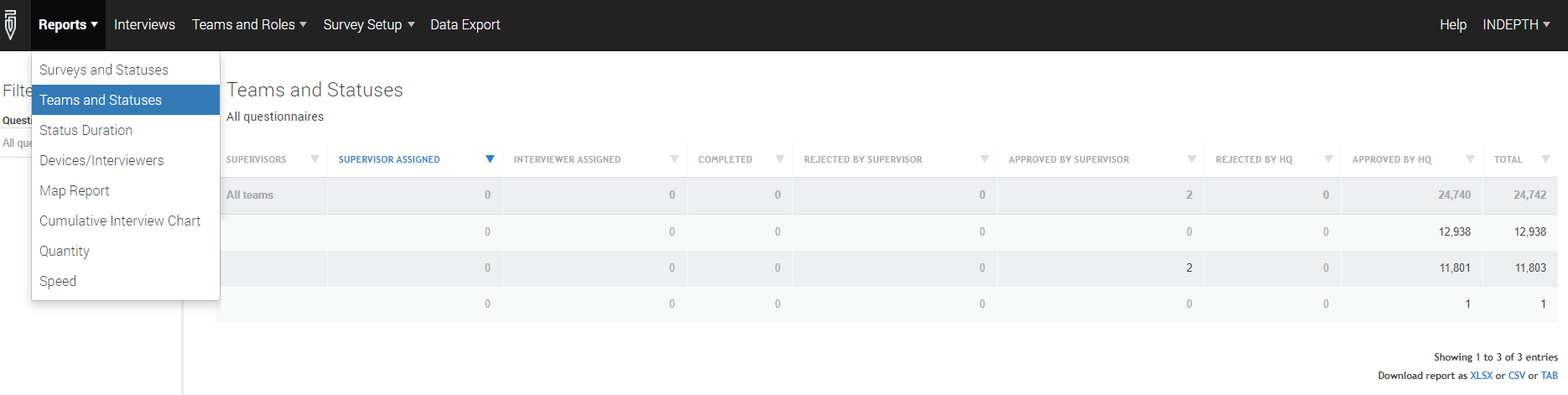


Reports – Status Duration


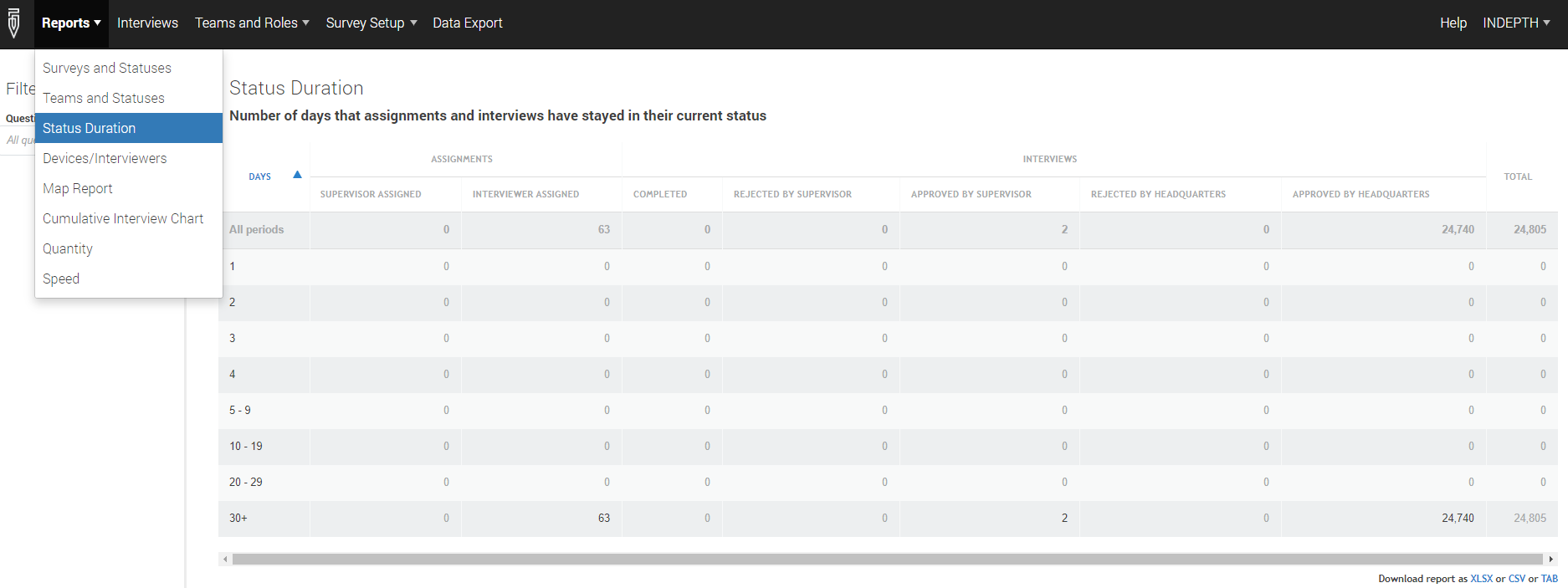


Reports – Devices/Interviewers


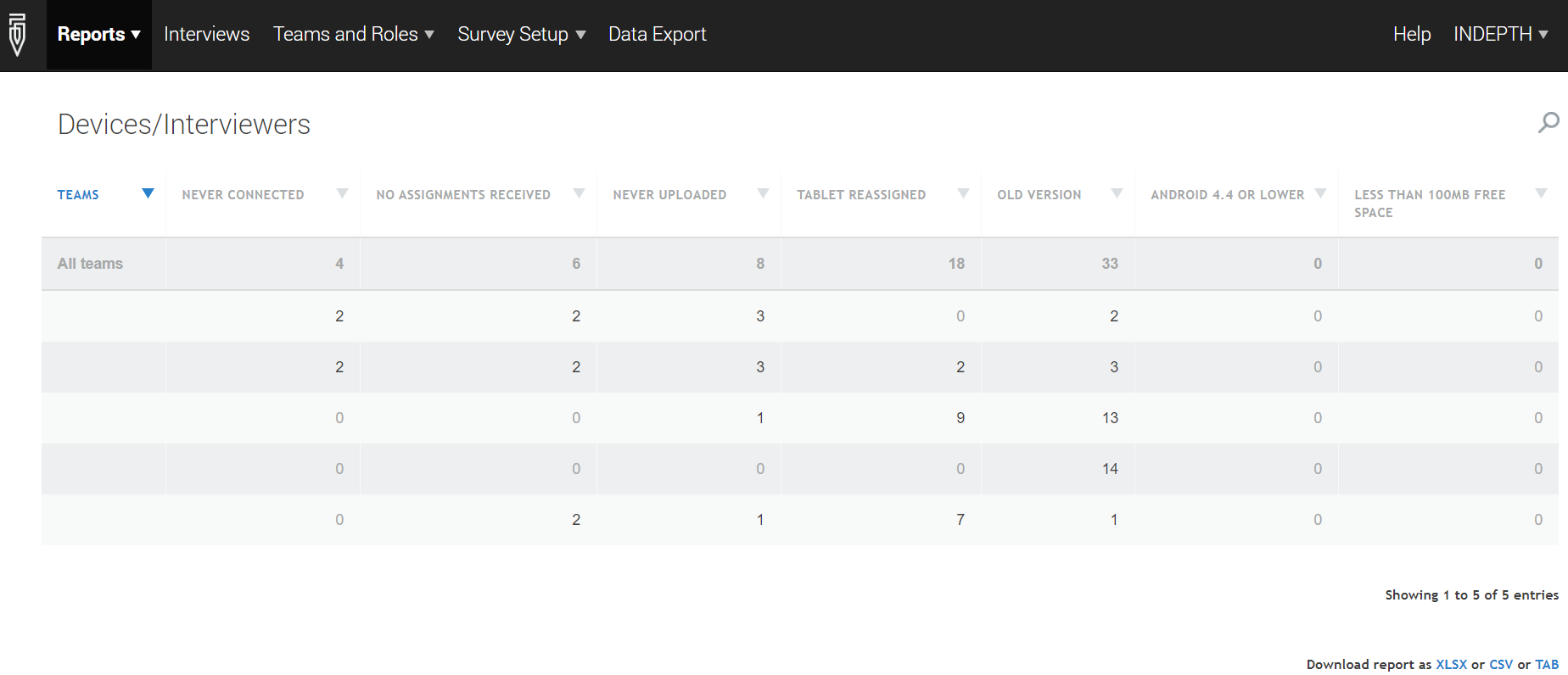


Reports – Filters


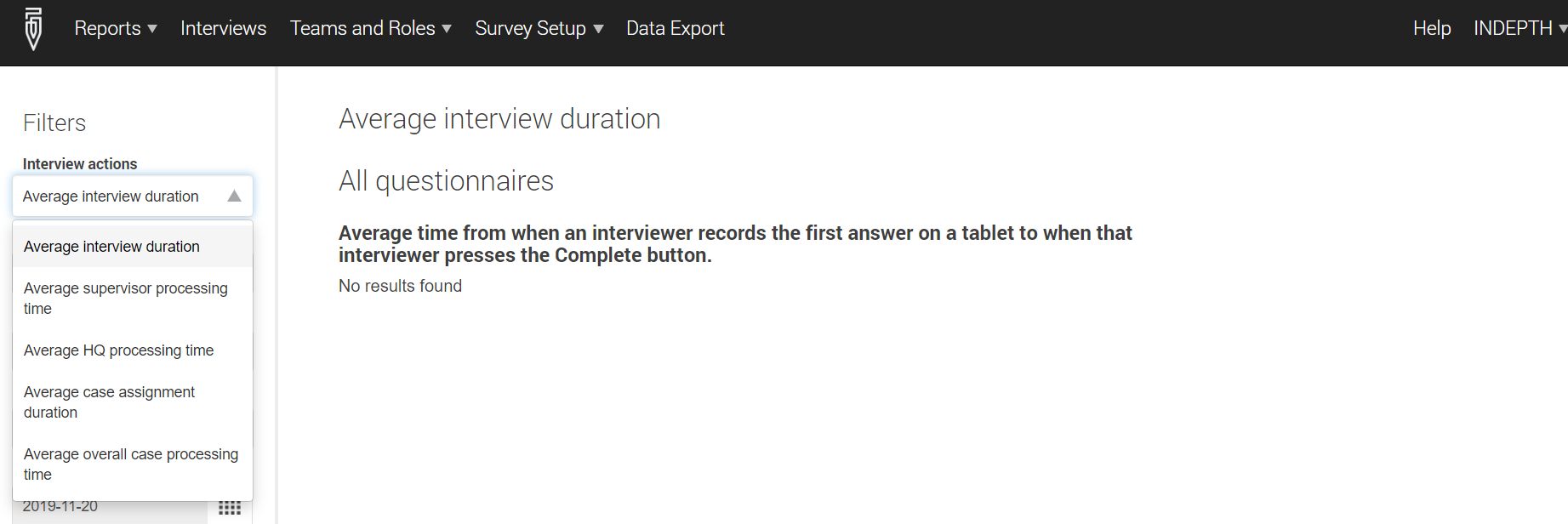


Reports – Map Report (a)


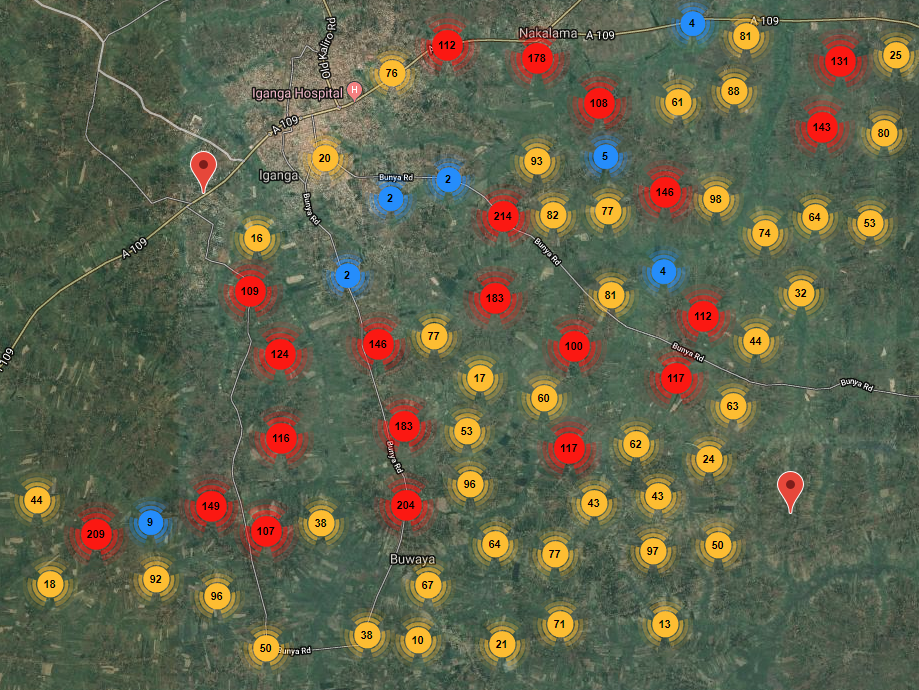


Reports – Map Report (b)


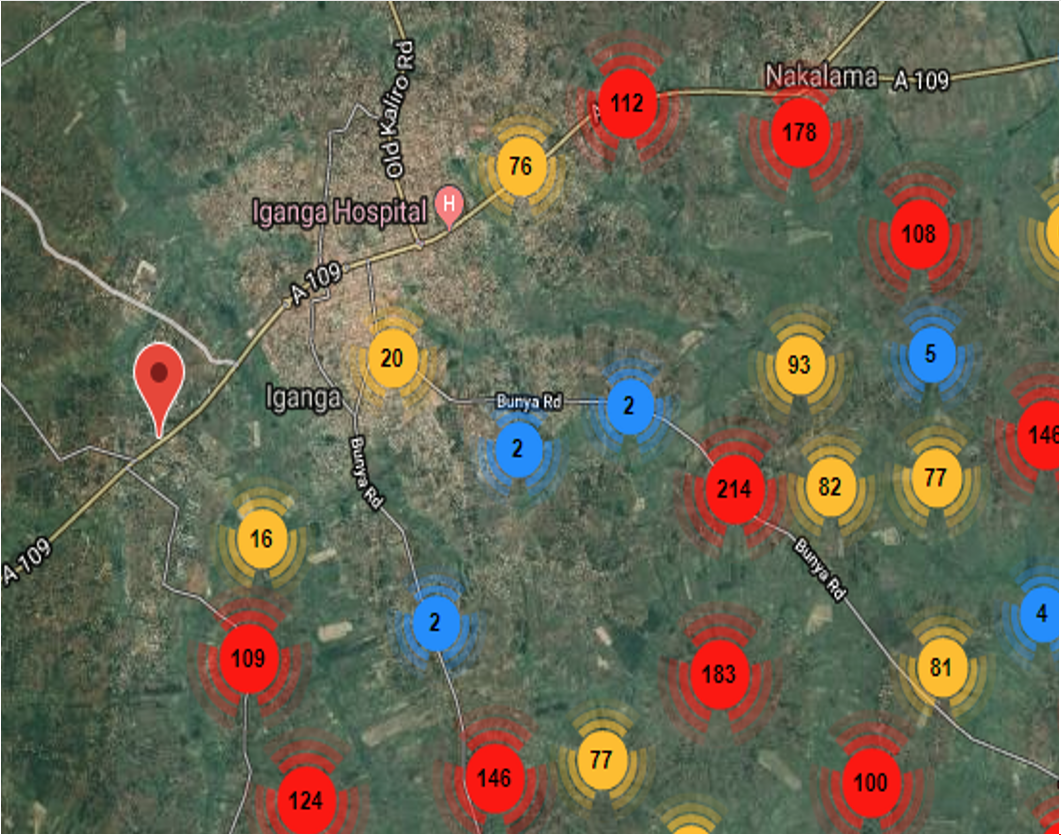


Reports – Cumulative Interview Chart (a)


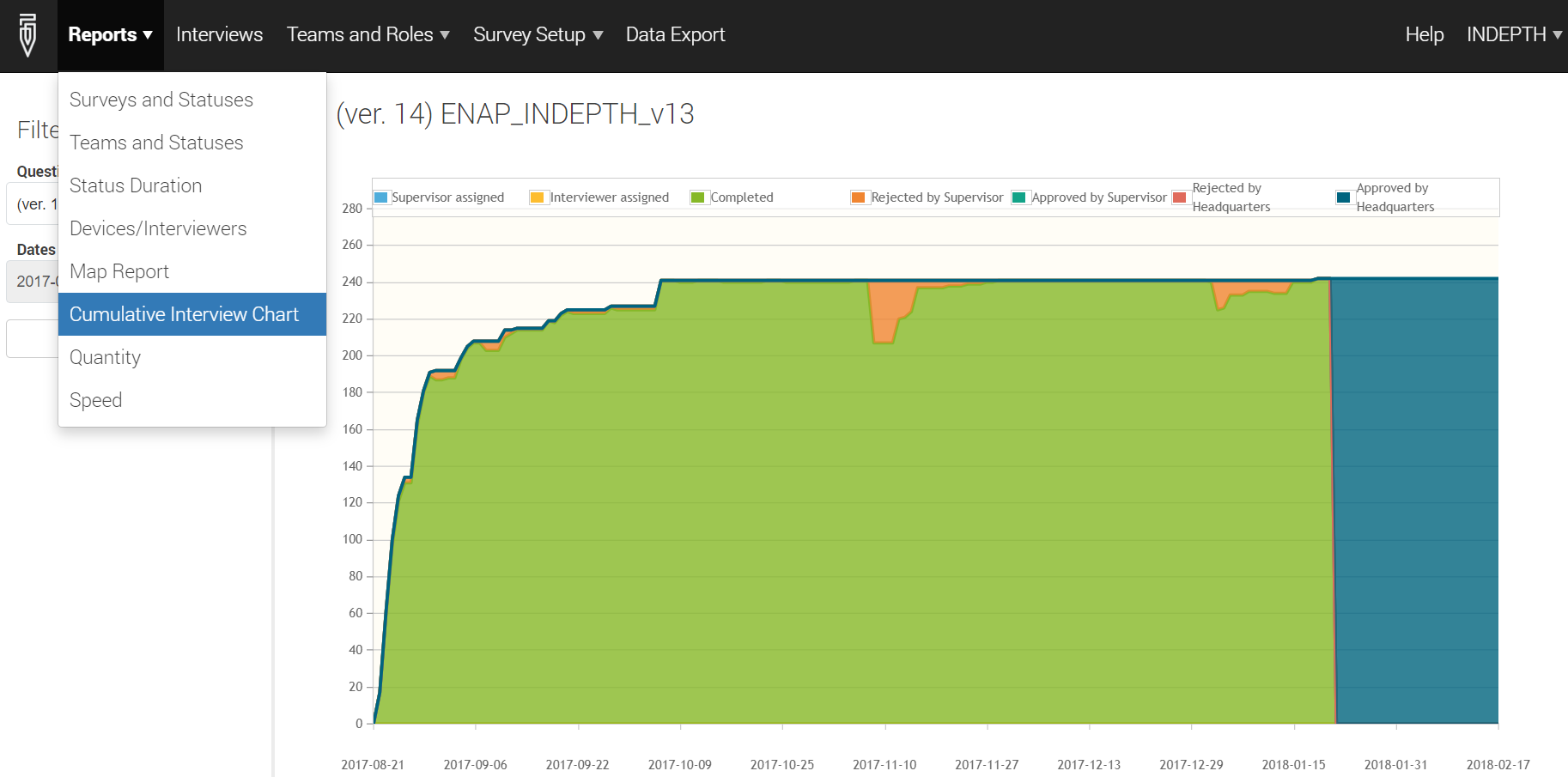


Reports – Cumulative Interview Chart (b)


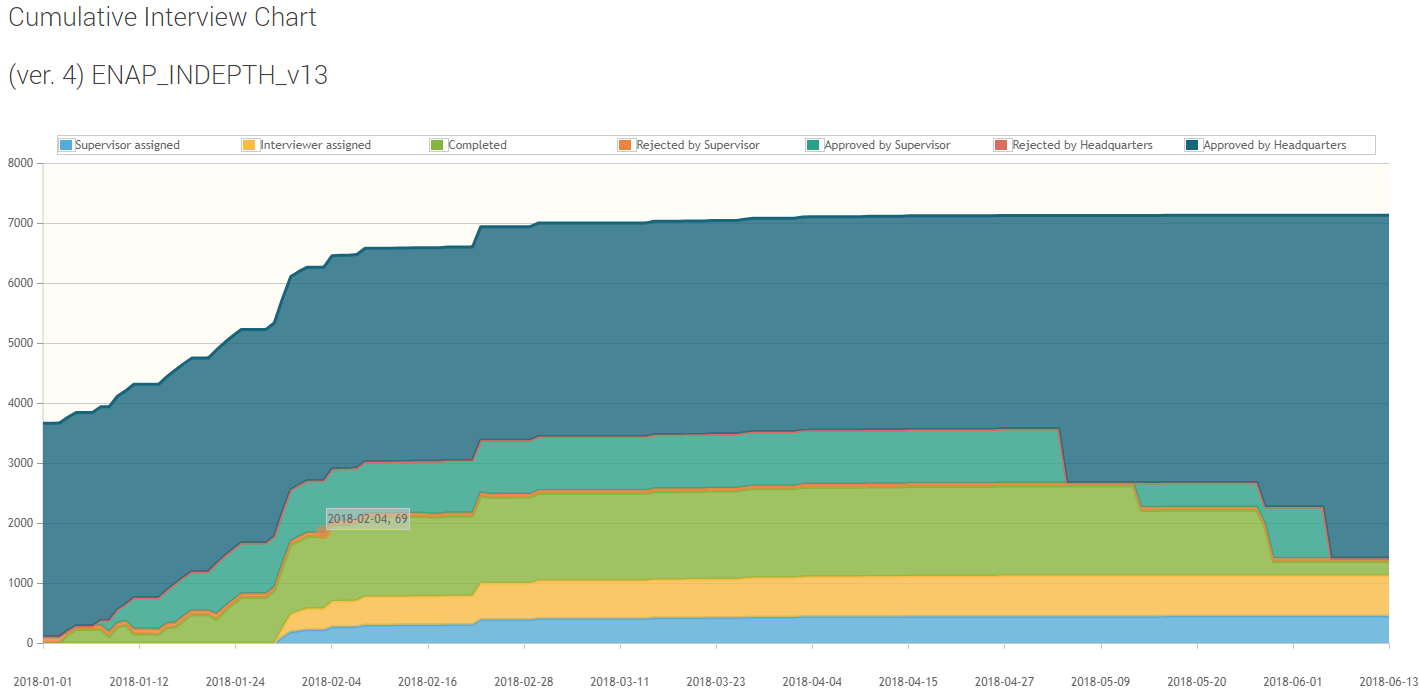


Reports – Cumulative Interview Chart (c)


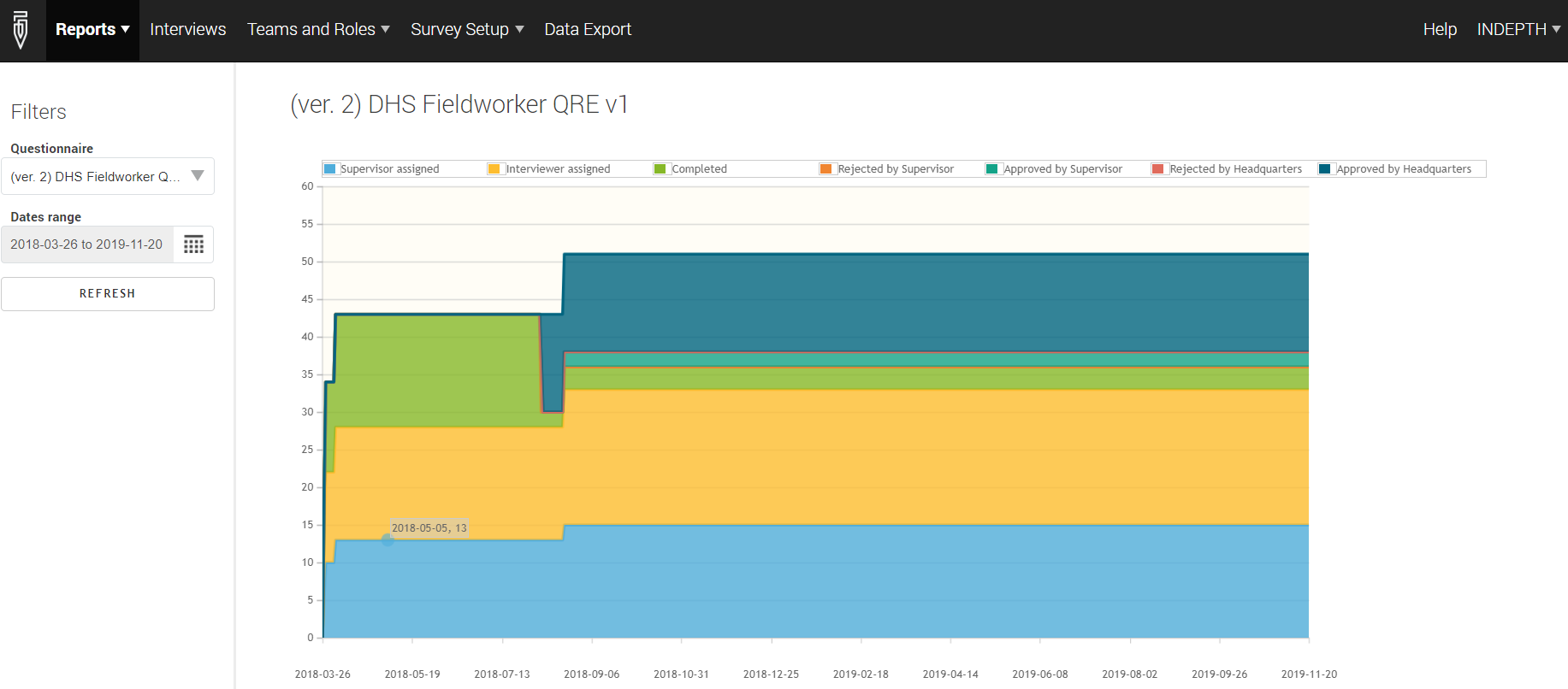


Reports – Quantity


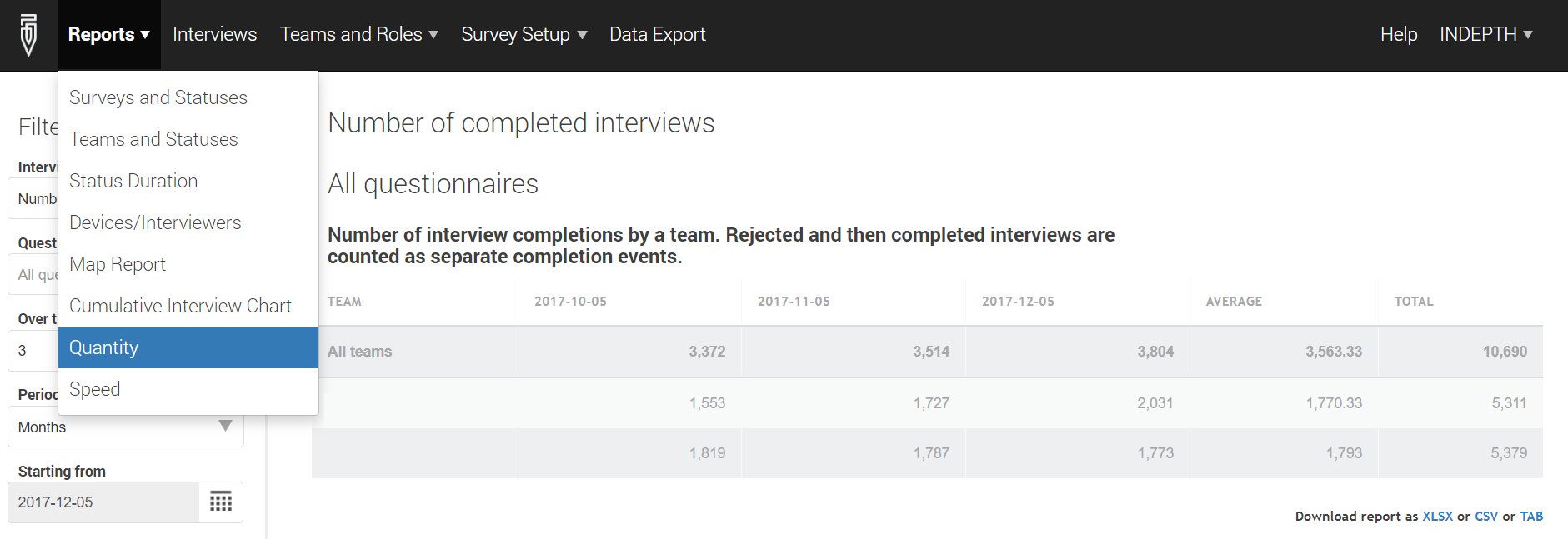


Reports – Speed


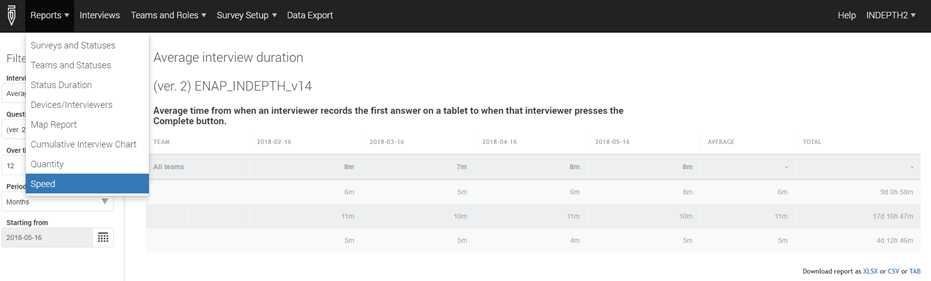


Interviews


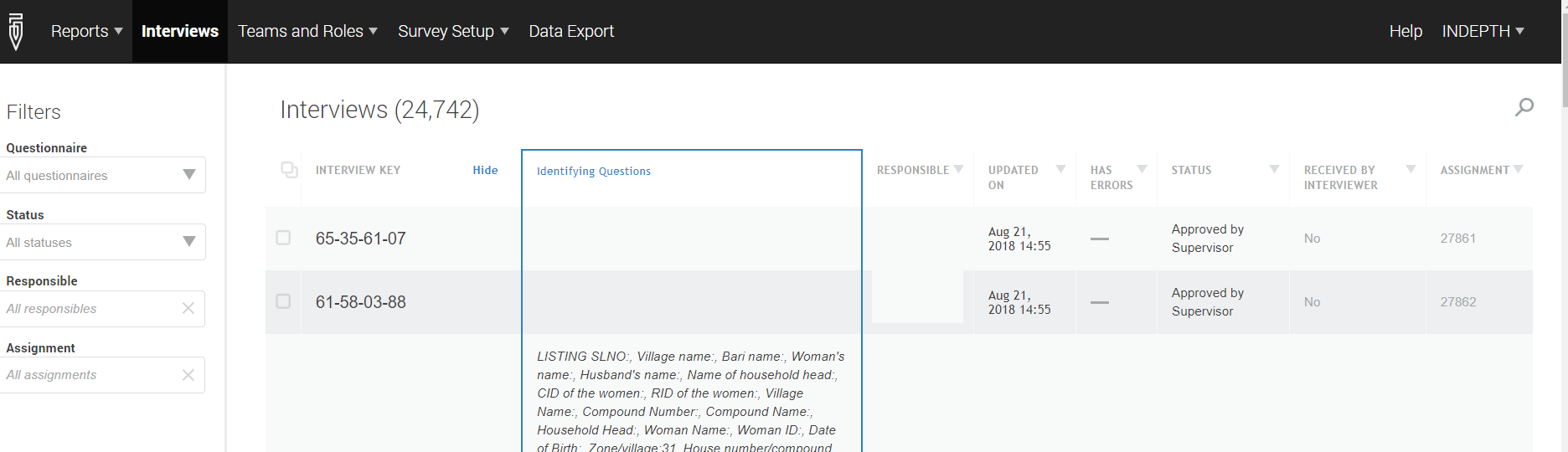


Teams and Roles – Headquarters


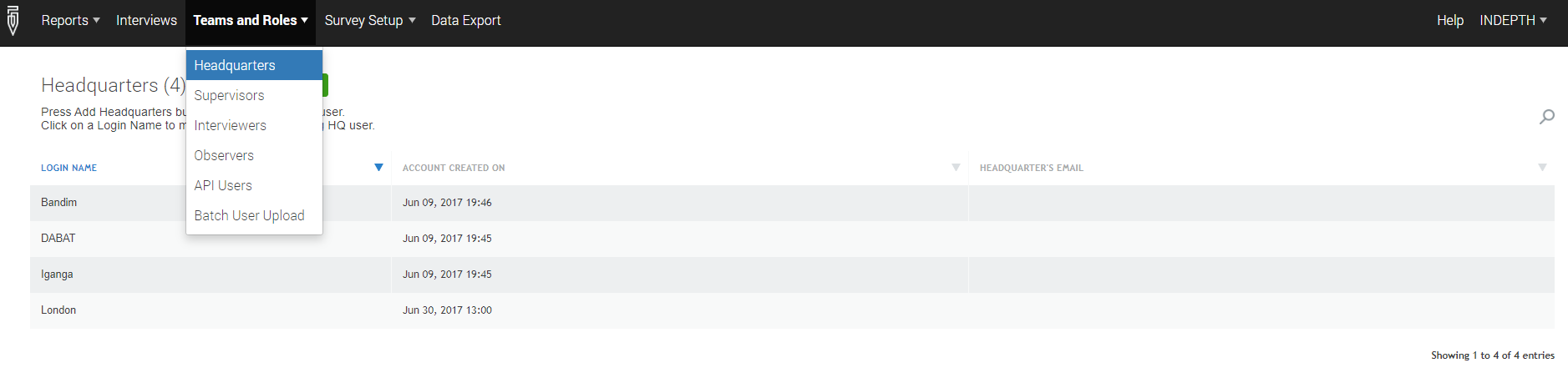


Teams and Roles – Interviewers


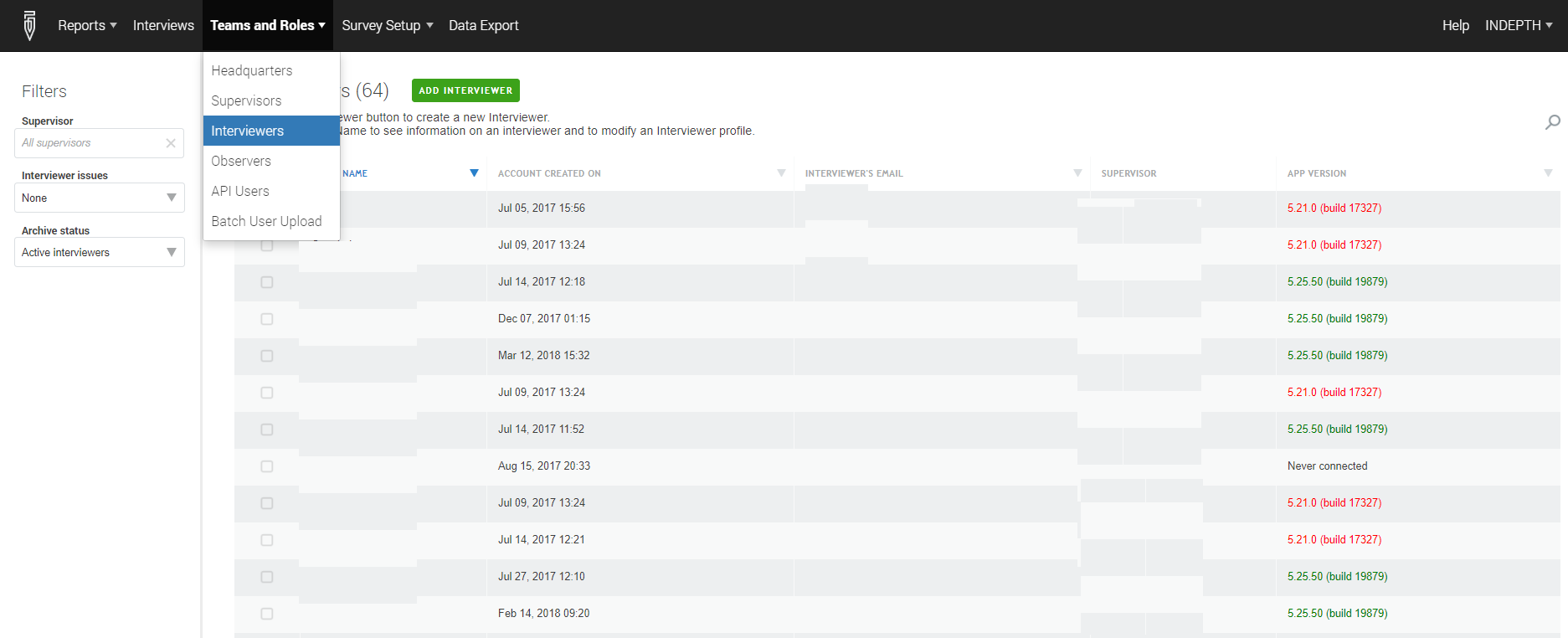


Teams and roles- detailed interview activity (incl. device information and synchronisation status)


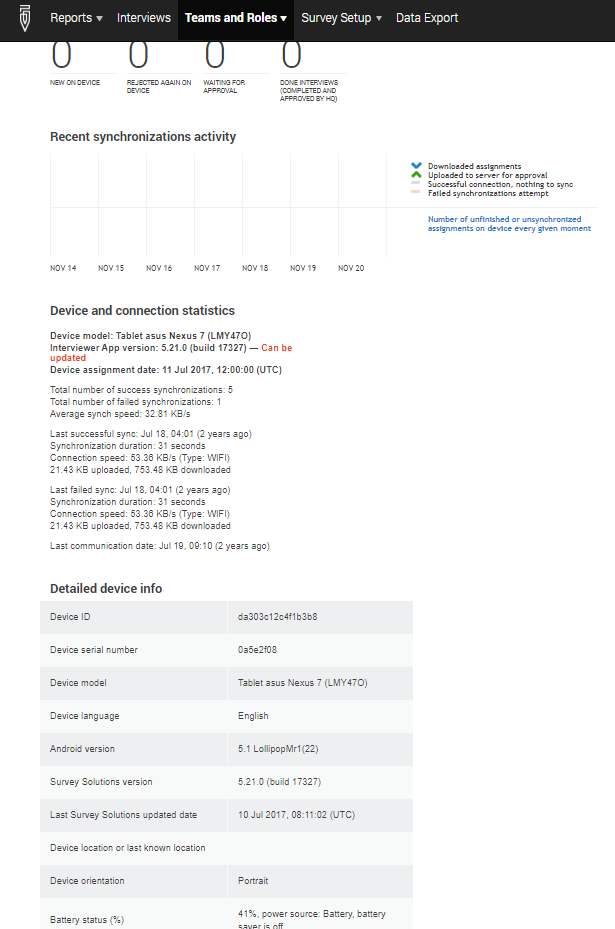


Survey setup – questionnaires


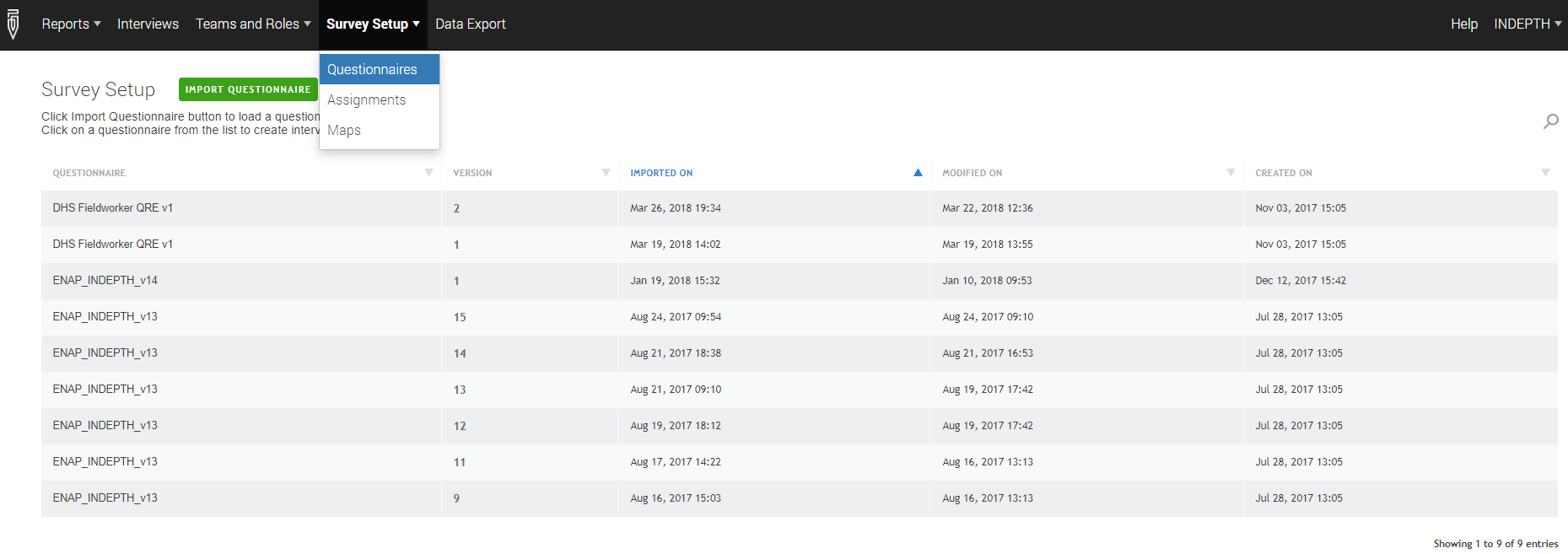


Survey setup – assignments


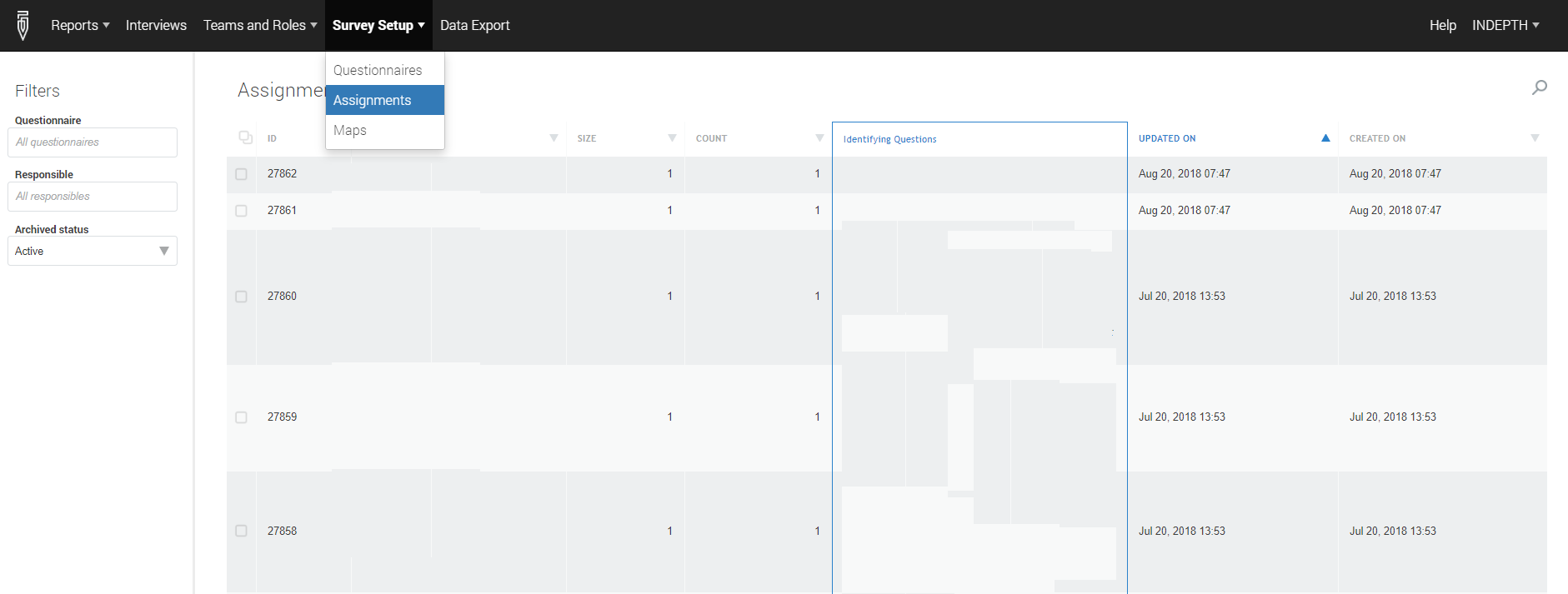


Data export


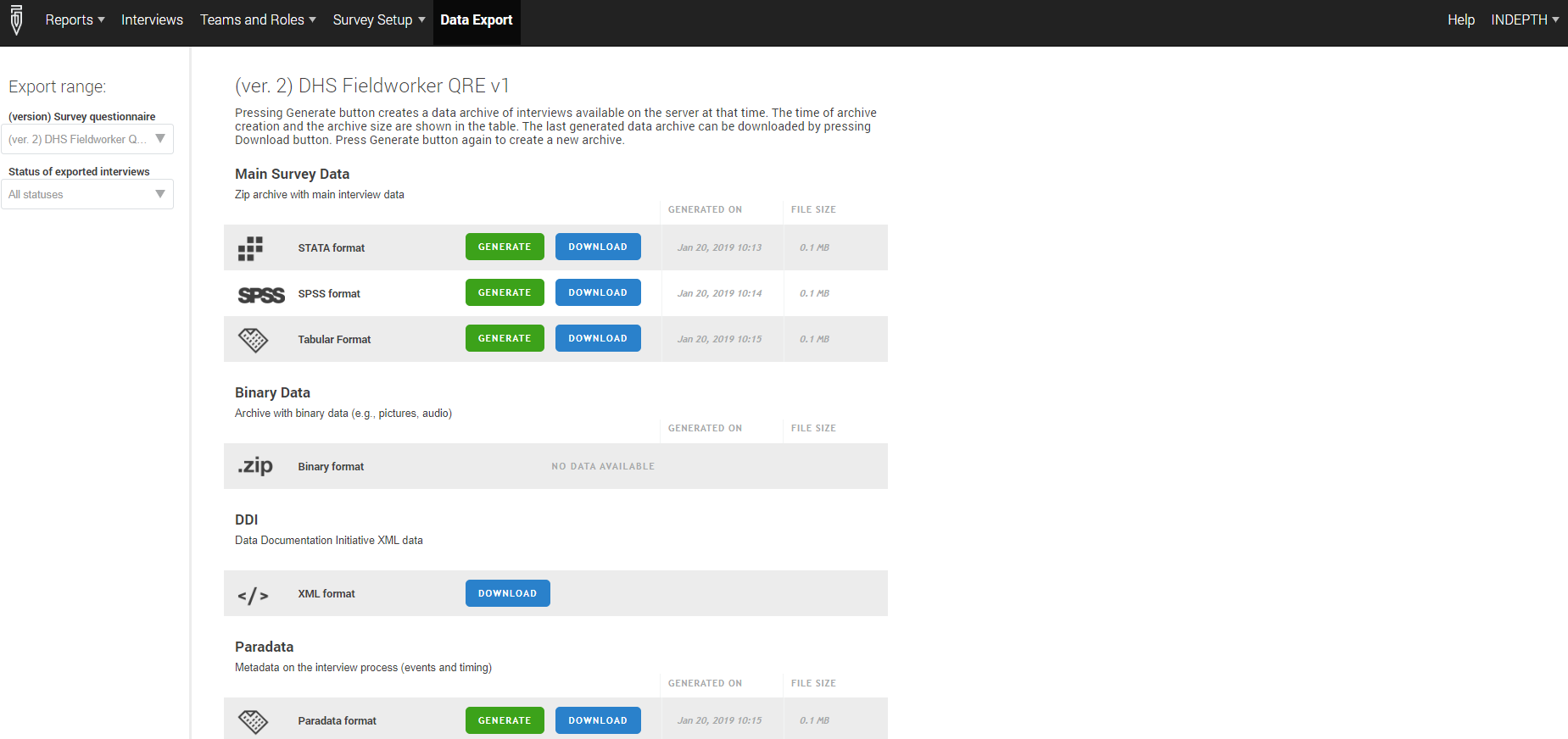


Interviewer application – main sections overview


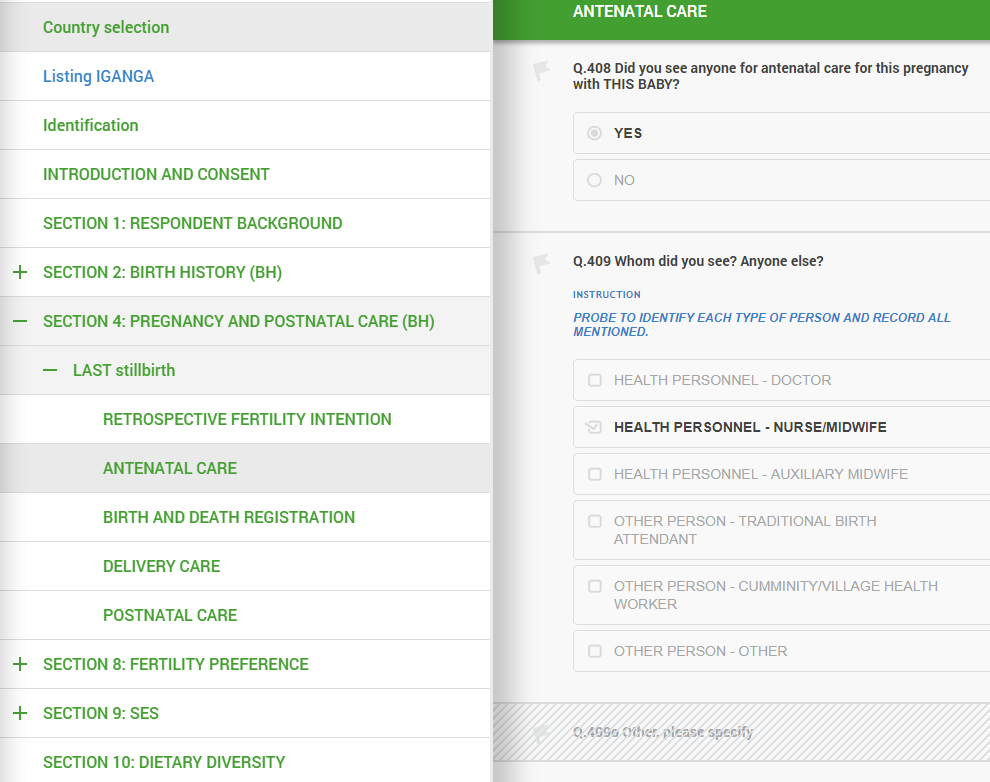

Supplement: Supplementary file 5 — Additional file 5. The World Bank Survey Solutions Headquarters overview. [file 12963_2020_226_MOESM5_ESM.docx]
