# Supplementary material for: Electronic data collection in a multi-site population-based survey: EN-INDEPTH study
Source: Popul Health Metr. 2021 Feb 8;19(Suppl 1):9. doi: 10.1186/s12963-020-00226-z (PMC7869201; doi:10.1186/s12963-020-00226-z)

# **Additional file 6: Overview of the ongoing routine and EN-INDEPTH study data collection at each site**


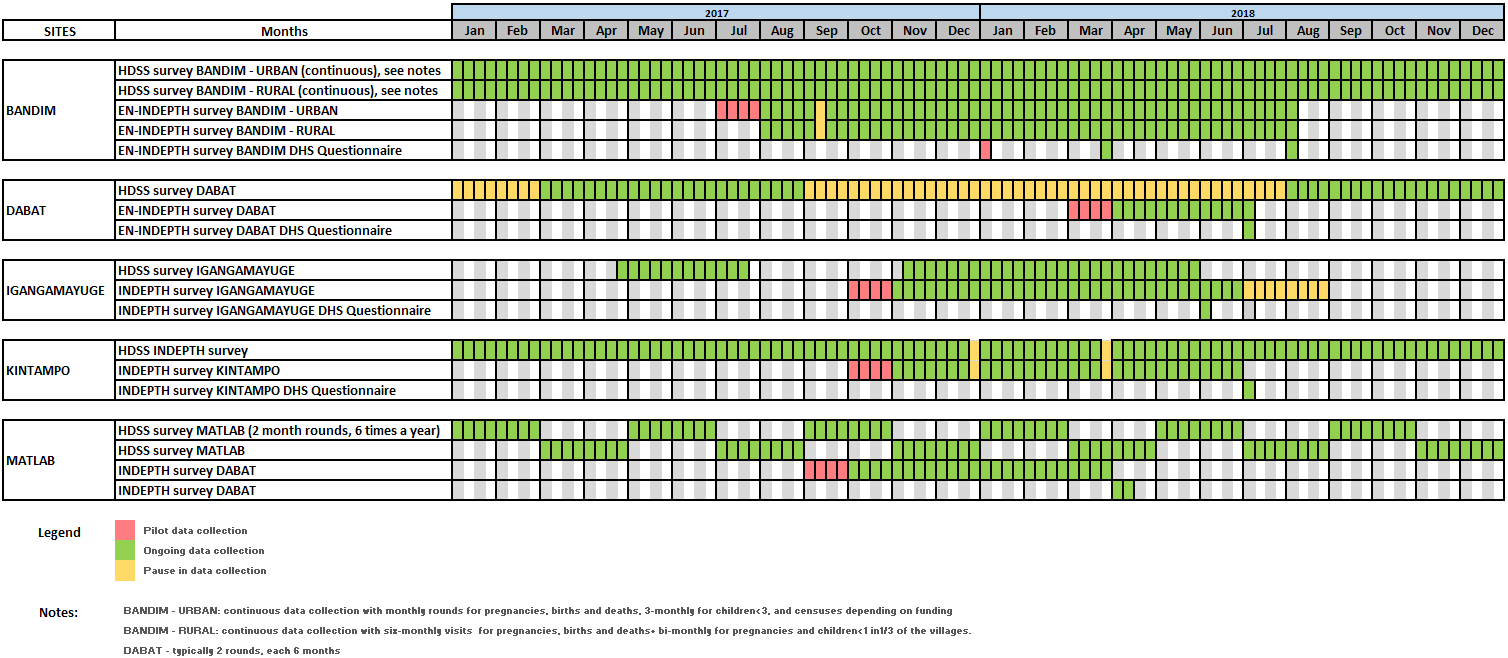

Supplement: Supplementary file 6 — Additional file 6. Overview of the ongoing routine and EN-INDEPTH study data collection at each site. [file 12963_2020_226_MOESM6_ESM.docx]
