# Supplementary material for: Electronic data collection in a multi-site population-based survey: EN-INDEPTH study
Source: Popul Health Metr. 2021 Feb 8;19(Suppl 1):9. doi: 10.1186/s12963-020-00226-z (PMC7869201; doi:10.1186/s12963-020-00226-z)
Supplement: Supplementary file 7 — Additional file 7. EN-INDEPTH overall progress site report. [file 12963_2020_226_MOESM7_ESM.docx]

# **Additional file 7: EN-INDEPTH overall progress site report**

| **Indicators** | **Variable name from Stata syntax** | 11.09.17 | 25.09.17 |
| --- | --- | --- | --- |
| ***Survey management*** |  |  |  |
| Total N of women eligible (HDSS) | *(to be copied form HDSS)* |  |  |
| Total N of women approached | Napproac |  |  |
| Number of women approached (identified) | Nidentified |  |  |
| Number of women approached (not identified) | Nnotid |  |  |
| Number of women that were not interviewed within 3 visits | Nnotint3v |  |  |
| Number of women consented | Nconsy |  |  |
| Number of women non-consented | Nconsn |  |  |
| ***Survey progress*** |  |  |  |
| Total N of women with BH | totBH |  |  |
| Total N of women with PH | totPH |  |  |
| Total N of births (BH+PH) between 2012-2017 | Nb1217 |  |  |
| Number of livebirths (BH+PH) between 2012-2017 | NlbBHPH |  |  |
| Number of stillbirths (BH+PH) between 2012-2017 | NsbBHPH |  |  |
| Number of neonatal deaths between 2012-2017 | NndBHPH |  |  |
| Total N women completed Section 4 (BH+PH) | Nsec4 |  |  |
| Total N women completed Section 4 (BH) | Nsec4BH |  |  |
| Total N women completed Section 4 (PH) | Nsec4PH |  |  |
| Total N women that should have had section 4 (BH+PH) | *After version 14 is updated* |  |  |
